# The Immune Microenvironment: New Therapeutic Implications in Organ Fibrosis

**DOI:** 10.1002/advs.202505067

**Published:** 2025-05-20

**Authors:** Xiangqi Chen, Chuan Wu, Fei Tang, Jingyue Zhou, Li Mo, Yanping Li, Jinhan He

**Affiliations:** ^1^ Department of Pharmacy, Institute of Metabolic Diseases and Pharmacotherapy, West China Hospital Sichuan University Chengdu 610041 China; ^2^ State Key Laboratory of Biotherapy, West China Hospital Sichuan University Chengdu 610041 China; ^3^ Center of Gerontology and Geriatrics, West China Hospital Sichuan University Chengdu 610041 China

**Keywords:** immune microenvironment, organ fibrosis, therapeutic targets

## Abstract

Fibrosis, characterized by abnormal deposition of structural proteins, is a major cause of tissue dysfunction in chronic diseases. The disease burden associated with progressive fibrosis is substantial, and currently approved drugs are unable to effectively reverse it. Immune cells are increasingly recognized as crucial regulators in the pathological process of fibrosis by releasing effector molecules, such as cytokines, chemokines, extracellular vesicles, metabolites, proteases, or intercellular contact. Therefore, targeting the immune microenvironment can be a potential strategy for fibrosis reduction and reversion. This review summarizes the recent advances in the understanding of the immune microenvironment in fibrosis including phenotypic and functional transformations of immune cells and the interaction of immune cells with other cells. The novel opportunities for the discovery and development of drugs for immune microenvironment remodeling and their associated challenges are also discussed.

## Graphical abstract

This review summarizes recent advances in understanding the immune microenvironment's role in fibrosis, focusing on phenotypic/functional alterations of immune cells and their dynamic interactions with other cellular constituents within tissues. The authors further explore therapeutic opportunities and challenges in targeting immune microenvironment reprogramming for antifibrotic drug development.

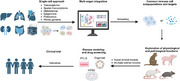

## 1 Introduction

Fibrosis is a chronic and progressive pathological process characterized by excessive deposition of extracellular matrix (ECM) components such as collagen and fibronectin due to dysregulated tissue repair response after injury. Temporary or minor injury results in proper deposition of ECM components to restore organizational structure and function; while, repetitive or severe injury results in dysregulated accumulation of ECM components and disrupted structure, which can lead to permanent scarring, even organ failure.^[^ ^1^ ^]^

**Figure 1:**
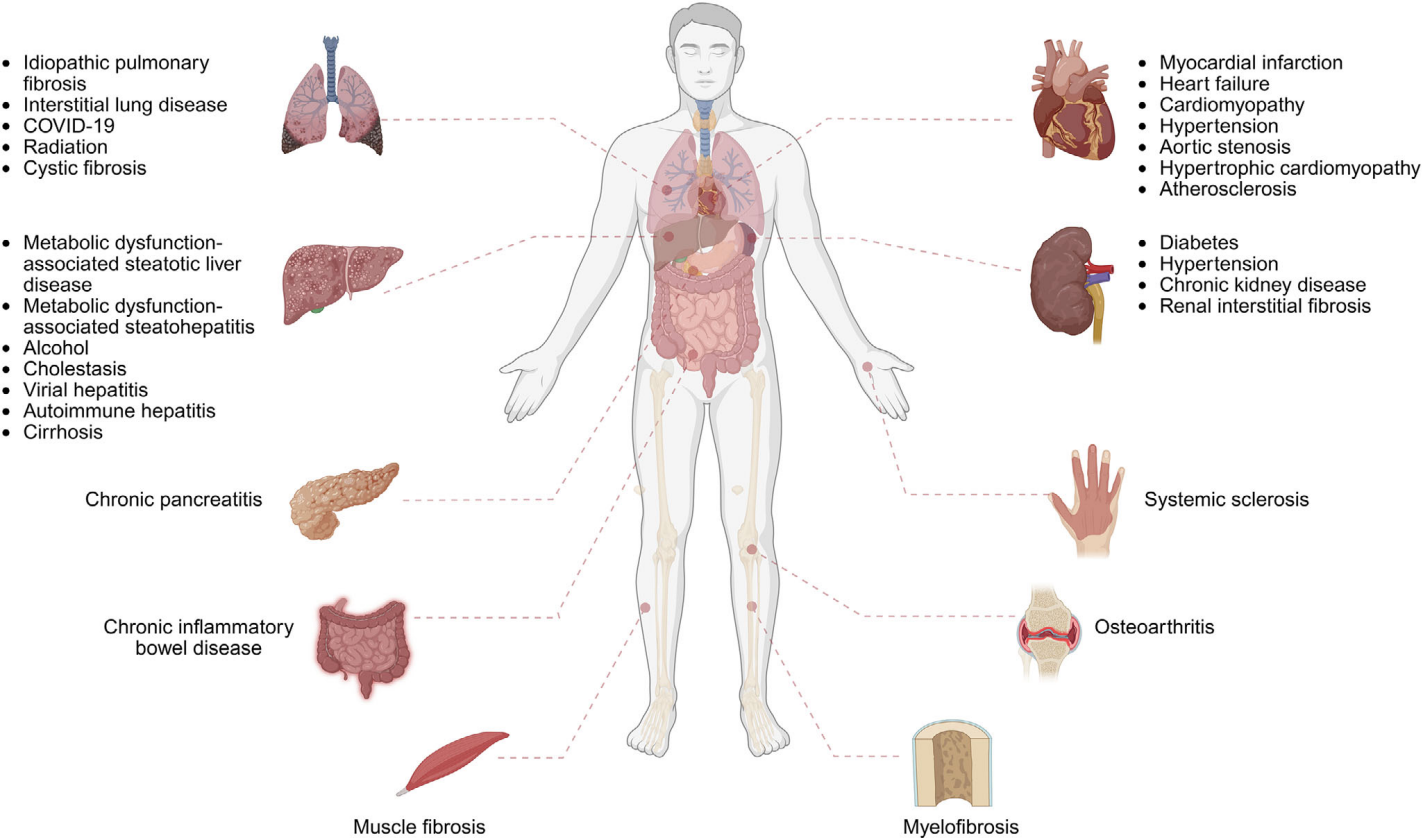
Common chronic human diseases associated with fibrosis are shown. Created with BioRender https://www.biorender.com/.

Fibrosis is a final, common pathological outcome of many chronic inflammatory diseases as seen in metabolic dysfunction‐associated steatohepatitis, idiopathic pulmonary fibrosis (IPF), heart failure, systemic sclerosis (SSc), keloids, and end‐stage renal disease with high incidence (**Figure** **1**). Statistics on the incidence of diseases associated with fibrosis of the liver, lung, kidney, skin, and heart show 4986 per 1 00 000 person years.^[^ ^2^, ^3^ ^]^ In addition, almost 45% of deaths in the industrialized countries are due to fibrosis.^[^ ^1^ ^]^ Although fibrosis is considered as the main cause of morbidity and mortality in many chronic inflammatory diseases, a growing number of therapeutic targets is being discovered and applied in clinical research including TGFβ inhibitors (Pirfenidone; GC1008; LY2383770), connective tissue growth factor inhibitor (Pamrevlumab; FG‐3019), angiokinase inhibitor (Nintedanib), THR‐β agonist (Rezdiffra), AMP‐activated protein kinase (AMPK) agonist (Resveratrol; PXL‐770), and PPARγ agonist (Caffeine plus chlorogenic acid; GI262570; Elafibranor).^[^ ^2^, ^4^, ^5^, ^6^, ^7^ ^]^ However, there's no effective treatment to reverse or prevent organ fibrosis including FDA‐approved Pirfenidone, Nintedanib, and Rezdiffra.

As the primary regulators of ECM production and remodeling, fibroblasts have become a focal point of research, aimed at identifying therapeutic drugs targeting the excessive deposition of the ECM in organ fibrosis. However, as important mediators of chronic inflammatory responses and participants in rapid response to wound healing processes, the regulatory role of immune cells in fibrosis, including communication with parenchymal cells, stromal cells, and other immune cells, has been greatly underestimated. A recent study has found that Siglec‐F^+^ neutrophils rapidly expand in the injured kidney with high expression of profibrotic inflammatory cytokines and collagen I that are essential for creating a profibrotic microenvironment in unilateral ureteral obstruction‐induced chronic kidney disease.^[^ ^8^ ^]^ Accumulation of eosinophils, macrophage, and T cells in fibrosis loci promotes the recruitment and activation of fibroblasts via releasing IL‐4, mEar1, and CCL1.^[^ ^9^, ^10^ ^]^ Interestingly, Chimeric antigen receptor (CAR) T‐cell therapy has exhibited astonishing therapeutic efficacy in cardiac fibrosis.^[^ ^11^ ^]^ It's now clear that almost all immune cells participate in organ fibrosis, and the increasing understanding of the molecular mechanisms governing this pathological process suggests the substantial therapeutic potential of targeting the immune microenvironment. This review will systematically summarize the roles of immune cells in fibrosis and the specific regulatory mechanisms from the perspective of the immune microenvironment, with the aim of identifying common pathways and targets to develop broadly antifibrotic drugs. Finally, the potential fibrosis therapeutic targets and therapeutic approaches of the immune microenvironment, as well as the challenges, are discussed.

## 2 The Immune Microenvironment

The immune system can be divided into innate immunity and adaptive immunity with different initiation times, immune cell members, and approaches, but exist crossover. Innate immunity, serving as the first line of host defense, rapidly responds to tissue injury and pathogen invasion. Its cellular components encompass granulocytes (neutrophils, eosinophils, and basophils), monocytes, macrophages, dendritic cells, innate lymphoid cells (ILCs), and unconventional T cells (γδ T cells, MAIT cells, and NKT cells). Upon activation by pathogen‐associated molecular patterns (PAMPs), damage‐associated molecular patterns (DAMPs), and metabolite‐derived danger signals, these innate immune cells can directly modulate the initiation and resolution of tissue inflammation or immune responses through phagocytosis and secretion of bioactive effector molecules.^[^ ^12^ ^]^ Alternatively, they may indirectly regulate these processes by activating APC‐mediated adaptive immune responses.^[^ ^13^ ^]^ Adaptive immunity mediated by T cells and B cells relies on the recognition of specific antigens presented by antigen‐presenting cells, such as dendritic cells and macrophages, via T cell receptors (TCRs) or B cell receptors (BCRs). Although adaptive immune responses are slower compared to innate immunity, they promote pathogen‐specific effector pathways, generate immunological memory, and regulate the persistence or resolution of chronic tissue inflammation as well as immune homeostasis through the release of bioactive effector molecules, thereby modulating tissue remodeling. Fibrosis represents a common endpoint of numerous chronic inflammatory diseases, characterized by a complex pathogenesis involving the interplay of multiple cell types. During tissue remodeling, tissue‐resident and infiltrating innate and adaptive immune cells respond to environmental cues by generating distinct bioactive effector molecules, which orchestrate heterocellular and homocellular communication between immune cells and other cellular components within the tissue.^[^ ^14^, ^15^ ^]^ These diverse molecules produced by immune cells govern whether the tissue progresses toward regeneration or fibrosis. Collectively, immune cells and their secreted mediators (e.g., cytokines, chemokines, exosomes, and metabolites) shape the immune microenvironment, thereby ultimately determining the balance between tissue repair and pathological fibrosis.

## 3 Macrophages

As an essential component of innate immunity, macrophages are the important regulator of the immune system and respond to diverse environmental signals. Macrophages reside in almost all body compartments. Macrophage derived from the yolk sac and fetal liver progenitors during embryogenesis performs as heterogenous and self‐renewing tissue‐resident populations, such as Kupffer cells (in liver), bronchoalveolar macrophages (in lung), microglial cells (in brain), and osteoclasts (in bone).^[^ ^16^ ^]^ Once inflammation and infection occur, huge numbers of inflammatory monocytes (macrophage precursors) from bone marrow are recruited via various chemokines and adhesion molecules to meet further demands. As highly plastic cells, monocyte‐derived macrophages and tissue‐resident macrophages undergo a phenotypic and functional transition in response to cytokines and growth factors derived from local tissue microenvironment after injury, which instruct macrophages to adopt pro/anti‐inflammatory, pro/anti‐fibrotic phenotypes. Macrophage dysfunction and failure of phenotype, function transitions contribute to abnormal repair—fibrosis, including uncontrolled pro‐inflammatory factors producing and persistent recruitment of inflammatory cells, activation or production failure of anti‐inflammatory or anti‐fibrosis macrophages, and abnormal communication between macrophages and other immune cells, endothelial cells, fibroblasts, stem cells, and epithelial cells.^[^ ^17^ ^]^ These characteristics of macrophages underscore their prominence in the initial focus of researchers investigating the process of wound healing. Therefore, we'll focus on the different roles of macrophages in fibrosis and emerging therapeutic opportunities below.

### 3.1 Heterogeneity of Macrophages in Fibrosis

Macrophages are remarkably heterogeneous, undergoing phenotypic and functional transition in response to surrounding different environmental cues. Generally, macrophages exist in two polarization states: 1) M1 macrophages, which are classically activated to perform pro‐inflammatory roles and polarized by lipopolysaccharide (LPS) and IFN‐γ and 2) M2 macrophages, which are alternative activated to perform anti‐inflammatory roles and polarized by IL‐4 and IL‐13.^[^ ^18^ ^]^ The roles of these two macrophage subsets in fibrosis are still controversial. Cu‐Zn‐SOD‐mediated H_2_O_2_ polarizes macrophages to an M2 phenotype via STAT6 to prompt the transcription of pro‐fibrotic factors and subsequent collagen deposition.^[^ ^19^ ^]^ Macrophages‐derived microRNAs have been shown to regulate macrophage polarization in fibrosis. MiR‐142‐5p and miR‐130a‐3p in macrophages, regulated by IL‐4 and IL‐13, modulate the activation of M2 macrophages to promote fibrogenesis.^[^ ^20^ ^]^ However, other results show that inducing M2 macrophages polarization can protect against fibrosis; while, polarizing toward M1 phenotype aggravates fibrosis.^[^ ^21^, ^22^ ^]^ Thus, by dividing macrophages into M1 and M2 subpopulations, more precise cell populations may be lost, leading to controversial conclusions. High‐throughput sequencing technologies are needed to classify and define macrophages for accurately targeting fibrosis‐related macrophage populations or investigating the function of specific subsets.

Here, delightful progress has been made in deciphering macrophages heterogeneity and capturing the phenotypic transformation of macrophages during fibrosis through scRNA‐seq, spatial transcriptomics, and RNA‐seq analysis. A profibrotic macrophages subset, CX3CR1^+^SiglecF^+^ macrophages with transitional gene expression profiles intermediate between monocyte‐derived and alveolar macrophages, was found by analyzing scRNA‐seq data of fibrotic lung samples in mice.^[^ ^23^ ^]^ The gene signatures of these transitional macrophages are also upregulated in IPF samples.^[^ ^23^ ^]^ Integration of human fibrotic liver and lung public scRNA‐seq datasets identifies a macrophage subset, CD9^+^TREM2^+^ macrophages, expressing *SPP1*, *GPNMB*, *FABP5*, and *CD63*, which are adjacent to activated mesenchymal cells and accumulate near scar loci.^[^ ^24^ ^]^ Blockade of GM‐CSF, IL‐17A, or TGF‐β1 suppresses fibrosis via inhibiting the differentiation of profibrotic CD9^+^TREM2^+^ macrophages.^[^ ^24^ ^]^ Profibrotic PLA2G7^high^ macrophages in a mixed M1/M2 state also highly express SPP1 and exist in both human pulmonary fibrosis patients and bleomycin‐treated mice; while, SPP1^high^MERTK^high^ macrophages are found in other scRNA‐seq research of IPF patients and they both contribute to myofibroblasts activation.^[^ ^25^, ^26^ ^]^ Further, the differentiation of profibrotic SPP1^+^ macrophages expanding in human chronic heart and kidney diseases is orchestrated by platelet‐derived CXCL4.^[^ ^27^ ^]^ Targeting SPP1^+^ macrophages in fibrosis may be an effective approach due to the majority of profibrotic macrophage subsets highly expressing SPP1.

Monocyte‐derived macrophages drive lung fibrosis via expressing various profibrotic genes that differ from tissue‐resident alveolar macrophages.^[^ ^28^ ^]^ In addition, monocyte‐derived macrophages highly expressing CSF1/CSF1R may constitute an autocrine M‐CSF/M‐CSFR loop to support their population and form a profibrotic circuit.^[^ ^29^ ^]^ Using anti‐CSF1R antibodies to specifically deplete interstitial macrophages (monocyte‐derived) can ameliorate lung fibrosis.^[^ ^30^ ^]^ Indeed, compared to alveolar macrophages (AMs), tissue‐infiltrating macrophages (IMs) have higher levels of CSF1R protein. Anti‐CSF1R antibody treatment ameliorates radiation‐induced lung fibrosis by selectively depleting profibrotic IMs, which drive myofibroblast activation via arginase‐1 upregulation; while, AMs depletion shows no therapeutic effect, revealing the potential mechanisms by which the CSF1R antibody exerts its effects.^[^ ^30^ ^]^ These results suggest the profibrotic role of monocyte‐derived macrophages in lung tissue, and the potent capacity of blocking CSF/CSFR signaling in fibrogenesis. Interestingly, in colorectal cancer, peritoneal‐resident immunosuppressive macrophages exhibit high expression levels of CSF1R, including SPP1^+^ macrophage populations.^[^ ^31^ ^]^ Moreover, anti‐CSF1R antibody treatment has been shown to deplete all pro‐inflammatory anti‐tumor macrophages.^[^ ^31^ ^]^ This observation raises the critical question of whether CSF1R‐targeting antibody therapy for fibrotic diseases could concurrently deplete pro‐fibrotic SPP1^+^ macrophages. Further experimental validation is warranted to evaluate the specificity and therapeutic efficacy of CSF1R blockade in modulating macrophage subpopulations, potentially offering novel insights into antifibrotic treatment strategies.

Anti‐fibrotic macrophages are found in nonalcoholic steatohepatitis (NASH) mice, including Trem2^+^ macrophages and Vsig4^+^ macrophages.^[^ ^32^, ^33^ ^]^ The restorative CD11B^hi^F4/80^int^Ly6C^lo^ macrophage subset found in the liver can secrete matrix metalloproteinases (MMPs) and activate the apoptotic pathway for fibrosis resolution.^[^ ^34^, ^35^ ^]^ Promoting the phenotypic switch from general pro‐fibrotic Ly6C^high^ macrophage to restorative Ly6C^low^ macrophage may be considered as a new fibrosis treatment. Although many fibrosis‐associated macrophage subpopulations have been identified, further studies need to delve into the kinetics of their transition in‐depth and whether specifically deleting one of them can inhibit fibrosis. Larger samples are needed for integrated analysis to avoid individual differences. Further, fibrotic samples need to be sequenced at different states to discover the turning points of phenotypic transformation and the cell subsets that play particular roles at certain times.

### 3.2 Pro‐Fibrotic Roles of Macrophages

**Figure 2:**
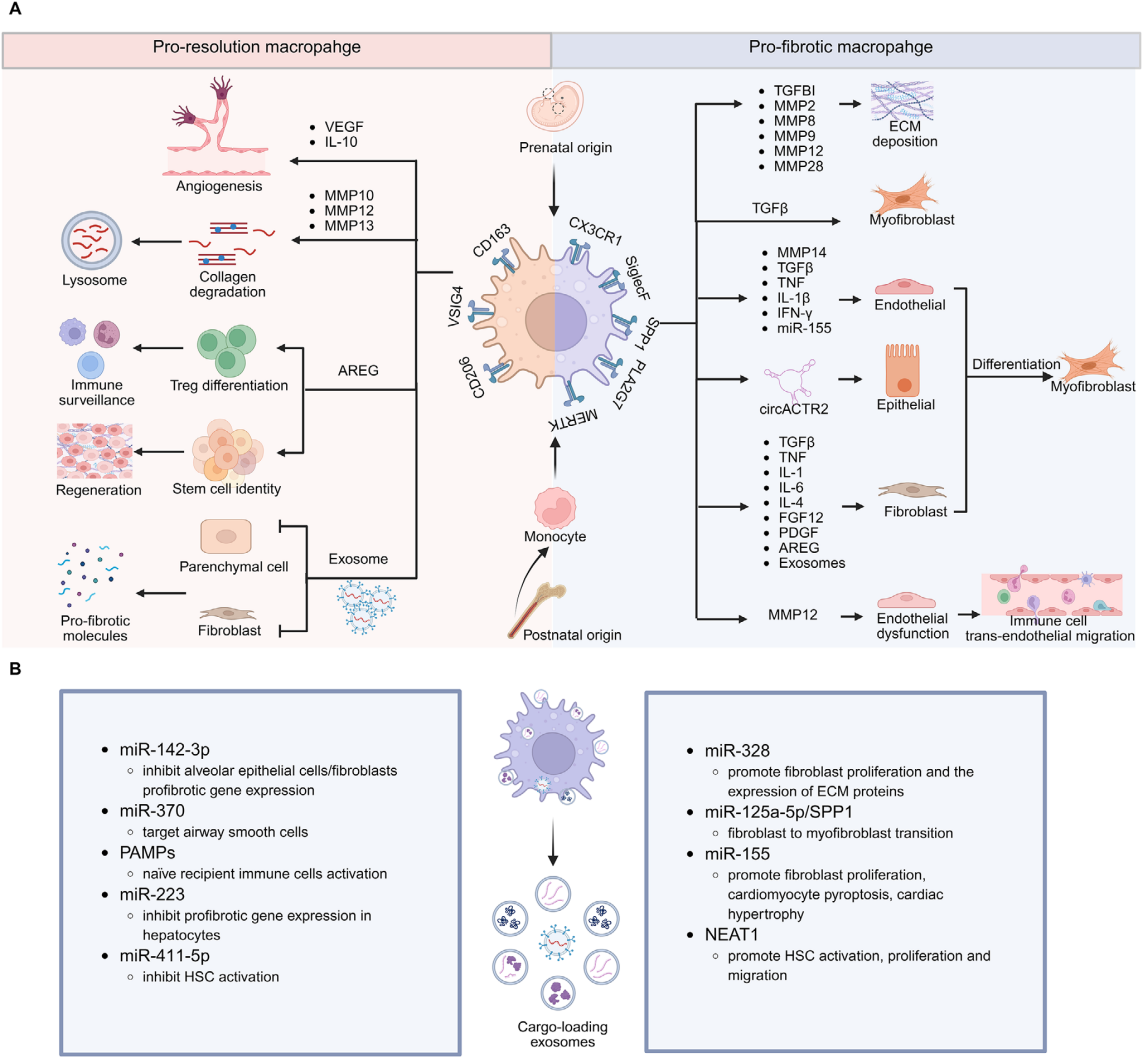
The dual role of macrophages in fibrosis: pro‐fibrotic drivers and resolution mediators. A) Tissue‐resident macrophages originate from the yolk sac and fetal liver precursors during embryogenesis. Bone marrow‐derived monocytes become the postnatal origin of macrophages. In response to environmental stimuli, macrophages undergo the phenotypic and functional transition to exert pro‐fibrotic or pro‐resolution characters. Macrophage‐released effector molecules including cytokines, MMPs, microRNA, and circRNA regulate epithelial/endothelial/fibroblast‐to‐myofibroblast transition. Macrophage‐produced MMPs (MMP2, MMP8, MMP9, MMP12, and MMP28) and TGFBI promote extracellular matrix overdeposition. Moreover, MMP12 production triggers endothelial dysfunction and failure of trans‐endothelial surveillance. On the contrary, pro‐resolution macrophage‐derived MMPs (MMP10, MMP12, and MMP13) induce collagen degradation, and AREG regulates Treg differentiation and stem cell identity to promote organ regeneration. In addition, macrophage‐released IL‐10 and VEGF mediate angiogenesis upon tissue injury. B) Macrophage‐secreted cargo‐loading exosomes participate in fibrosis. Created with BioRender https://www.biorender.com/.

Macrophages have been shown to produce multiple cytokines to regulate fibroblasts and myofibroblasts activation, migration, proliferation, ECM synthesis, and further inflammatory cell recruitment, including TNFα, IL‐1, IL‐6, IL‐4, TGFβ, FGF12, and PDGF.^[^ ^23^, ^36^, ^37^, ^38^, ^39^ ^]^ The crosstalk between macrophages and fibroblasts leads to fibrosis, which was further maintained by contracting fibroblasts‐generated deformation fields in fibrillar collagen matrix to attract macrophages over distance.^[^ ^40^ ^]^ Moreover, macrophages may release TNFα and IL‐1β to activate alveolar epithelial cells, which produce numerous cytokines and chemokines (such as TNFα, IL‐1β, and MCP‐1), to recruit more leukocytes migration to the fibrotic foci.^[^ ^41^ ^]^ In SSc‐associated interstitial lung disease, SPP1^+^ macrophages upregulate chemokine CCL18 to attract immune cells and stimulate collagen overproduction.^[^ ^42^ ^]^ Macrophages are one of the origins of Amphiregulin (AREG), which plays important roles in wound healing, including promoting fibroblast migration and collagen synthesis.^[^ ^43^, ^44^ ^]^ In the case of chronic lung inflammation, macrophages‐produced AREG promotes fibroblast motility and proliferation and induces myofibroblast differentiation indirectly.^[^ ^45^ ^]^ However, recent studies show that macrophage‐derived AREG also induces pericyte into myofibroblast differentiation to contribute to collagen synthesis for wound healing^[^ ^46^ ^]^ and increases regulatory T (Treg) cells, preserving epithelial stem cells for immune suppression and tissue regeneration.^[^ ^47^ ^]^ These results indicate that AREG released by macrophages is essential for immune regulation, tissue repair, and fibrogenesis. Targeting macrophages‐derived AREG at different stages of disease will have different effects on fibrosis. As a phagocyte, macrophages can clear apoptotic cells and necrotic debris to contribute to inflammation resolution for tissue repair. However, monocyte‐derived macrophages produce TGF‐β‐induced (TGFBI) to downregulate MMP14 levels in fibroblast, leading to final collagen deposition, following ingestion of apoptotic cells,^[^ ^48^ ^]^ which indicates the balance of macrophages ingesting apoptotic cells is crucial to determine whether to promote wound healing or fibrosis (**Figure** **2**A).

In addition to regulating the pro‐fibrotic phenotype, functional transition of fibroblasts, and ultimately excessive extracellular matrix deposition by releasing cytokines, macrophages are also capable of releasing cytokines to hijack epithelial–mesenchymal transition (EMT) and endothelial‐to‐mesenchymal transition (EndoMT) process to contribute to fibrogenesis in pathological conditions. Previous study shows that macrophage‐derived circular RNA ACTR2 (circACTR2) mediates EMT of tubular epithelial cells in a paracrine manner through the production of IL‐1β in renal fibrosis.^[^ ^49^ ^]^ EndoMT is another way to generate myofibroblast, which plays an important role in fibrosis‐related diseases. Macrophages have also been shown to contribute to EndoMT via MMP14/TGFβ after MI.^[^ ^50^ ^]^ Exosomal miR‐155 released by M1 macrophages impairs vascular endothelial cells and promotes EndoMT upon spinal cord injury.^[^ ^51^ ^]^ TGFβ, TNF, IL‐1β, and IFN‐γ are potential cytokines produced by macrophages to stimulate EndoMT.^[^ ^52^ ^]^ Conversely, CD206^+^ anti‐inflammatory macrophages have been shown to contribute to maintaining endothelial identity and inhibit EndoMT by producing VEGF and IL‐10.^[^ ^53^, ^54^ ^]^ Thus, cytokines released by macrophages help us schematize complex cellular interactions during fibrosis and provide new insights into targeting cytokines.

Myofibroblasts are the major cell type in ECM production and the final cellular effector of fibrosis, which can be derived from various cell types, including epithelial cells via EMT, endothelial cells via EndoMT, fibroblasts, and pericytes. Notably, a new myofibroblast genesis has been discovered that macrophages, derived from circulating monocytes, can trans‐differentiate to myofibroblast via macrophage‐to‐myofibroblast transition (MMT). Over the past years, numerous studies have shown the existence of MMT, co‐expression monocyte/macrophage, and fibroblast/myofibroblast markers in various fibrosis conditions including renal,^[^ ^55^ ^]^ cardiac,^[^ ^56^ ^]^ and pancreas^[^ ^57^ ^]^ fibrosis, which constitutes a reasonable therapeutic target to prevent and improve fibrosis disease. In kidney, MMT triggers the TGFβ1/Smad3 signaling pathway and IL‐4 (produced by NKT cells) signaling pathway, which enhance the MMT process in turn.^[^ ^58^ ^]^ Approximately 90% P2Y12^+^ macrophages have been shown to undergo MMT by co‐expressing myofibroblast markers and further validating by scRNA‐seq. Targeting P2Y12 can block TGF‐β/Smad3‐mediated MMT and progressive renal fibrosis.^[^ ^59^ ^]^ Two targets of Smad3 in renal fibrosis, Pou4f1^[^ ^55^ ^]^ and proto‐oncogene tyrosine protein kinase Src,^[^ ^60^ ^]^ were proved to be crucial downstream regulators of MMT. In addition, Caspase‐11/GSDMD‐dependent extracellular traps (NETs) formation can trigger the MMT process to exacerbate renal fibrosis in obstructive nephropathy.^[^ ^61^ ^]^ In pancreatic ductal adenocarcinoma, oxidative stress has been shown to be able to activate p38‐MAPK to induce myofibroblast marker expression in monocyte/macrophage,^[^ ^57^ ^]^ indicating MMT may contribute to pancreas fibrosis governed by the p38‐MAPK axis. Moreover, M2 macrophages have two subsets: 1) pro‐fibrotic M2a subsets, promoting MMT and 2) anti‐fibrotic M2c subsets, promoting tissue repair. Excessive TGF‐β stimulation promotes M2a macrophages undergoing MMT; while, moderate TGF‐β stimulation promotes the M2c phenotype transformation.^[^ ^62^ ^]^ Thus, circulating monocytes, macrophages expressing myofibroblast markers, and the mechanism involved in MMT could provide new insights for antifibrotic therapies. Further study is needed to address MMT functions and the microenvironment cues for this process (Figure 2A).

Moreover, macrophages can secret MMPs to regulate the ECM deposition and intercellular communication in fibrosis. In IPF patients, macrophages‐derived MMP8 and MMP28 are increased.^[^ ^63^, ^64^ ^]^ In radiation‐induced pulmonary fibrosis, senescent macrophage‐derived Mmp2, Mmp9, and Mmp12 are also increased, which might trigger fibrotic phenotype transition in fibroblasts.^[^ ^37^ ^]^ Further, macrophage‐derived MMP12 causes endothelial dysfunction, including the decrease of viability, migration, and trans‐endothelial resistance.^[^ ^65^ ^]^ Further investigation is required to elucidate the underlying mechanisms by which MMPs function as a mediator of cell‐to‐cell communication in fibrosis.

Macrophages not only release various cytokines and MMPs to participate in fibrosis but also secrete exosomes, which carry a variety of biomolecules including nucleic acids, proteins, lipids, and other bioactive substances. This result suggests the immunomodulation role of macrophages via releasing exosomes in fibrosis. Previous study shows that M2 macrophages‐released exosomes, overexpressing miR‐328, are shown to aggravate pulmonary fibrosis via FAM13A.^[^ ^66^ ^]^ Exosomal miR‐125a‐5p derived from macrophages stimulates fibroblast to myofibroblast transdifferentiation in silicosis.^[^ ^67^ ^]^ During cardiac injury, activated macrophages secrete miR‐155‐enriched exosomes to exacerbate fibroblast proliferation and inflammation.^[^ ^68^ ^]^ In hepatic fibrosis, macrophages can release endosomal NEAT1^[^ ^69^ ^]^ to contribute to HSCs proliferation and migration by promoting the TGF‐β1/Smad signaling pathway; while, delivering exosomes loaded with anti‐fibrotic biomolecules represents a promising therapeutic strategy for tissue fibrosis. Thus, understanding their precise roles in the progression of fibrosis helps us take effective measures to prevent it (Figure 2B).

### 3.3 Pro‐Resolution Roles of Macrophages

Despite the prevailing notion that macrophages predominantly contribute to fibrosis, a growing body of evidence indicates that these cells may paradoxically play a role in establishing an anti‐fibrotic immune microenvironment by releasing pro‐resolution effector molecules. Macrophages‐produced MMP10^[^ ^70^ ^]^ and MMP13^[^ ^71^ ^]^ promote ECM degradation and moderate scar formation. Macrophages‐derived exosomes transfer miR‐142‐3P to alveolar epithelial cells and fibroblasts to inhibit profibrotic gene expression and thereby ameliorate pulmonary fibrosis.^[^ ^72^ ^]^ M2 macrophage‐derived exosomal miR‐411‐5p inhibits HSC activation.^[^ ^73^ ^]^ The production of miR‐223‐enriched macrophage exosomes triggered by myeloid cell‐specific IL‐6 signaling can reduce profibrotic transcriptional activator with PDZ‐binding motif expression in hepatocytes; and then, attenuate NASH.^[^ ^74^ ^]^ In addition, exosomes secreted by macrophages, containing PAMPs, lead to naïve recipient immune cell activation,^[^ ^75^ ^]^ which is important for immune surveillance (Figure 2A,B).

## 4 Dendritic Cells

As a versatile and efficient antigen‐presenting cell, dendritic cell (DC) plays critical roles in innate immune cell activation and triggering adaptive immune response. DCs are generally divided into four subsets:1) type 1 classical DCs (cDC1); 2) type 2 classical DCs (cDC2); 3) plasmacytoid DCs (pDC); and 4) monocyte‐derived DCs (mo‐DC).^[^ ^76^ ^]^ pDCs primarily produce type 1 interferon following recognition of virus or pathogen‐derived nucleic acids; while, classical DCs and mo‐DCs uptake, process, and present antigens to T cells. Upon inflammation or in mucosal tissues, monocytes can differentiate into DCs to regulate innate and adaptive immunity. In addition to homeostasis, pathogen infection, autoimmune diseases, and cancer, increasing evidence indicates that DCs participate in fibrosis. In the steady state, DCs remain immature and suppress adaptive immune response, including through triggering T cell apoptosis and Treg cell differentiation. After acute infection or sterile injury, DCs recruit and activate innate immune cells such as granulocytes or macrophages; whereas, persistent injury or failure to clear pathogens by innate immune cells may lead to the persistence of DAMPs or PAMPs, which trigger DCs to activate T cells and subsequent adaptive immune response.^[^ ^77^ ^]^ The disorder of one of these steps and DC abnormalities may lead to the occurrence of tissue injury and fibrosis.

**Figure 3:**
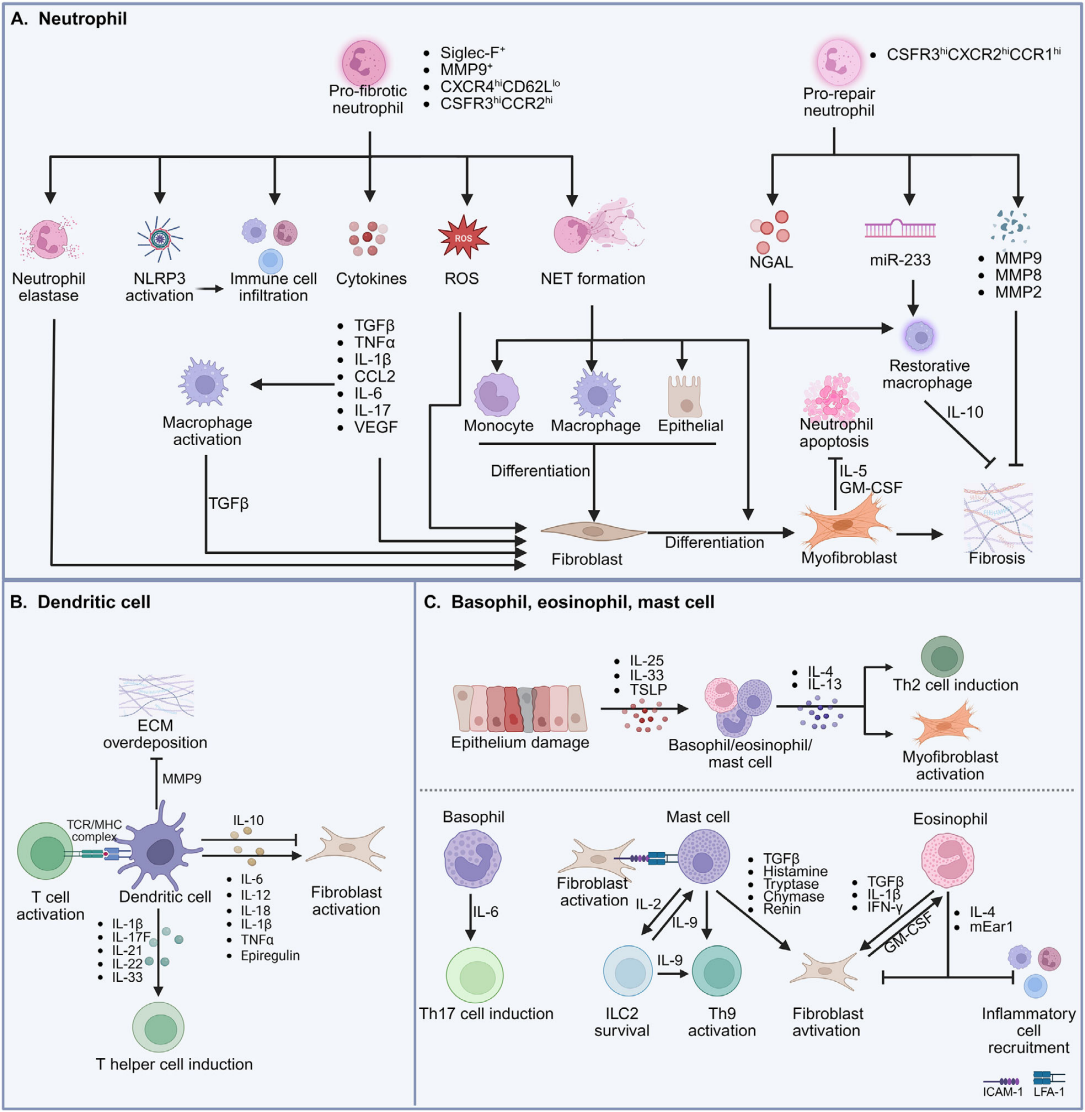
A) Neutrophils in fibrosis: a double‐edged sword for therapeutic targeting. Circulating neutrophils infiltrated in tissues perform pro‐fibrotic (Siglec‐F^+^/MMP9^+^/CXCR4^hi^CD62L^low^/CSFR3^hi^CCR2^hi^) or pro‐repair (CSFR3^hi^CXCR2^hi^CCR1^hi^) functions in response to different environmental cues. Neutrophils release various cytokines to regulate fibroblasts and macrophage activation, such as TGFβ, TNFα, IL‐1β, and CCL2. The production of ROS and neutrophil elastase promotes fibroblast differentiation. The activation of NLRP3 inflammasome in neutrophils further facilitates immune cell infiltration. Pro‐fibrotic neutrophil‐generated NET causes monocyte, macrophage, epithelial, and fibroblast differentiation; while, pro‐repair neutrophils produce NGAL and miR‐233 to promote macrophage polarization to restorative phenotype. In addition, neutrophil‐derived MMPs mediate ECM degradation to prevent overdeposition. B) Dendritic cells in fibrosis. Dendritic cells release pro‐fibrotic factors such as IL‐6, IL‐12, and IL‐18 to promote fibroblast activation and anti‐fibrotic factors such as IL‐10 and MMP9 to inhibit fibroblast activation and ECM deposition. In addition to inducing T cell activation by presenting antigen peptides through major histocompatibility complex (MHC) molecules, dendritic cells can also produce cytokines (IL‐1β, IL‐17F, IL‐21, IL‐22, and IL‐23) that promote the differentiation of helper T cells. C) Basophils, eosinophils, and mast cells in fibrosis, when epithelial damage, basophil, eosinophil, and mast cells can produce IL‐4 and IL‐13 to induce myofibroblast activation and Th2 cell induction. In addition to continuous coactivation through heterotypic cell connections, mast cells can also release TGFβ, histamine, and granule contents to activate fibroblasts. Mast cell‐secreted IL‐2 induces ILC2 survival, which produces IL‐9 to continuously activate Th9 cells and mast cells. Further, mast cells‐released IL‐9 triggers Th9 activation in turn. Basophils‐released IL‐6 promotes Th17 cell induction. For eosinophils, they can produce IL‐4 and mEar1 to regulate inflammatory cell infiltration and fibroblast activation. Fibroblast‐secreted GM‐CSF induces eosinophil activation and prolonged survival, which result in various pro‐fibrotic cytokine productions, such as TGFβ, IL‐1β, and IFN‐γ. Created with BioRender https://www.biorender.com/.

Previous study shows that TNFα‐producing CX3CR1^+^ mo‐DCs contribute to hepatic inflammation during steatohepatitis progression.^[^ ^78^ ^]^ In addition, pDCs may play a key role in skin fibrosis, and targeting pDCs alleviates SSc,^[^ ^79^, ^80^ ^]^ indicating the pro‐fibrotic role of pDCs and mo‐DCs. In SSc patients, mo‐DCs with altered function drive profibrotic inflammation by producing type 2/type 17 cytokines, IL‐33, and triggering aberrant T cell polarization.^[^ ^81^ ^]^ Increasing evidence shows that the overproduction of CXCL4 in pDCs from patients with SSc triggers the overproduction of IFNα.^[^ ^82^ ^]^ Using monoclonal BDCA2 antibodies to inhibit type I interferon secretion by pDCs can significantly ameliorate skin fibrosis, suggesting the crucial role of pDC‐derived IFNα in SSc development.^[^ ^79^ ^]^ Among DCs‐derived cytokines, IL‐6 and TNFα are known to stimulate HSC activation;^[^ ^83^ ^]^ while, MMP9 promotes fibrosis regression, and immature DCs‐derived IL‐10 negatively regulates HSC activation in liver.^[^ ^84^, ^85^ ^]^ Several studies show that IL‐22 protects liver fibrosis and promotes organ‐level repair through STAT3 activation.^[^ ^86^, ^87^ ^]^ As a predominant producer of IL‐22, DCs may contribute to tissue repair without scarring via releasing IL‐22. Moreover, as a DCs‐derived EGFR ligand, epiregulin activates fibroblasts to contribute to ECM overproduction in skin and lung fibrosis.^[^ ^88^ ^]^ Activated CD1c^+^ DC in the presence of the proximal epithelial cells undergoing ferroptosis shows NLRP3 inflammasome signaling activation, which is responsible for the release of pro‐inflammatory cytokines including IL‐1β and IL‐18 and is pivotal for tubulointerstitial fibrosis and inflammation.^[^ ^89^ ^]^ Thus, the immaturation and immunosuppression of DCs could be considered as anti‐fibrotic approaches. Exhilarating, Kinsenoside, which can alleviate liver fibrosis, suppresses the maturation of DCs, induces PD‐L1 expression, and leads to the interception of CD8^+^ T‐cell activation and a decrease in the production of proinflammatory cytokines, such as IL‐12, which is correlated with HSC activation (**Figure** **3**B).^[^ ^90^ ^]^

The anti‐fibrotic roles of DCs have also been noted. Atrophy of the dermal white adipose tissue is a feature of skin‐fibrosing disease, scleroderma; while, adipose tissue contains adipose‐derived mesenchymal stromal cells (ADSCs) with regenerative and reparative functions to improve skin fibrosis. Interestingly, DCs expressing LTβ are shown to be supportive for ADSCs survival via activating LTβR/β1 integrin pathway on ADSCs,^[^ ^91^ ^]^ indicating a pro‐repair function of DCs. Further, DCs emerge as anti‐fibrogenesis players during liver fibrosis by enhancing the expression of Flt‐1 to counteract the pro‐fibrotic role of VEGF to inhibit angiogenesis.^[^ ^92^ ^]^ All of the above results suggest the modulation role of DCs, both anti‐ and pro‐fibrotic, but it is still not clearly understood. Further studies are needed to discriminate the DCs from other myelomonocytic; so that, exploring the role of DCs, even specific subsets, in fibrosis‐related diseases becomes more practical.

## 5 Neutrophils

Neutrophils, the most abundant leukocytes in the blood, have long been viewed as the first line of cellular response for host defense and final effectors of the innate immune system during acute inflammation. As a granulocyte, neutrophils are characterized by their primary role in the clearance of extracellular pathogens and cell debris by multiple means, including phagocytosis, degranulation, and releasing neutrophil NETs. The functions and characteristics of neutrophils suggest their vital roles in the inflammatory response rapid activation and resolution following tissue damage. Further, it is clear now that neutrophils participate in pathogen clearance, inflammation (both acute and chronic), wound healing, autoimmunity, and cancer, signaling almost all immune cells to participate in the establishment of the basic network of immunity.^[^ ^93^, ^94^, ^95^ ^]^ In this review, the roles of neutrophils in unresolved chronic inflammation‐induced fibrosis are highlighted.

### 5.1 Heterogeneity of Neutrophils

Neutrophils are highly plastic cells. The development of single‐cell technology has significantly enhanced our comprehension of the functional and phenotypic heterogeneity of neutrophils based on transcriptional and epigenetic properties. Once in tissues, neutrophils perform diversified roles in certain tissues by phenotypic and functional changes in response to signals generated by the tissue microenvironment.^[^ ^96^ ^]^ The CXCR4^high^CD62L^low^ neutrophils (senescent phenotype) are accumulated in chronic obstructive pulmonary disease (COPD) and cystic fibrosis (CF) patients, which display higher proclivity to form NETs.^[^ ^97^, ^98^ ^]^ In the obstructed kidney of unilateral ureter obstruction (UUO)‐treated mice, MMP9^+^ neutrophils are essential for the establishment of profibrotic immune microenvironment through promoting neutrophils infiltration and other inflammatory cell accumulation.^[^ ^99^ ^]^ In another study, a large and rapidly expanding population of neutrophils expressed Siglec‐F are found in UUO‐injured kidney, which is derived from conventional neutrophils in the blood. Siglec‐F^+^ neutrophils create a profibrotic environment to activate fibroblasts by releasing TGFβ, TNFα, and IL‐1β to contribute to renal fibrosis.^[^ ^8^ ^]^ Neutrophils with Siglec‐F^high^ signature are also found in cardiovascular inflammation, including atherosclerotic vessels and myocardial infarction‐injured heart, using single‐cell transcriptomics combined with cell surface epitope detection sequencing.^[^ ^100^ ^]^ These findings suggest the possibility that targeting the profibrotic Siglec‐F^+^ neutrophil subpopulation may ameliorate fibrosis in multiple organs. Single‐cell RNA sequencing (scRNA‐seq) also identifies two distinct neutrophil clusters that respond to pressure overload‐induced heart injury, CSFR3^hi^CCR2^hi^ neutrophils, and CSFR3^hi^CXCR2^hi^CCR1^hi^ neutrophils. The CSFR3^hi^CCR2^hi^ neutrophils may represent a pro‐inflammatory subset; while, CSFR3^hi^CXCR2^hi^CCR1^hi^ neutrophils represent a pro‐repair subset,^[^ ^101^ ^]^ even with the growing numbers of studies attempting to depict the neutrophil heterogeneity in health and chronic inflammation conditions, and to describe disease‐associated, specialized subpopulations. However, whether such new neutrophil subsets actually exist is unclear. In consideration of the sensitivity to perturbation, manipulations in vitro, and the character of short‐lived, it's hard to catch the bona fide neutrophil clusters (including numbers, proportions, and transcriptional level). Thus, it's essential to develop normative protocols for the isolation of pure neutrophils to perform a precise definition of the phenotypes and functions using second/third‐generation sequencing or other high‐throughput sequencing. Thereby, neutrophil subsets with specific functions can be used as novel diagnostic markers and therapeutic targets in fibrosis‐related diseases.

### 5.2 Pro‐Fibrotic Roles of Neutrophils

Neutrophils transcribe little after leaving from the bone marrow; once activated, they experience a transcribe burst to synthesize signaling molecules to communicate with multiple cells, including themselves, to perform pro‐remodeling (pathological) functions. IL‐8 secreted from fibroblasts promotes the migration and production of CCL2 by neutrophils to affect the activity of macrophages, which highly express TGFβ to regulate fibroblast‐to‐myofibroblast transdifferentiation.^[^ ^102^ ^]^ The imbalance between NK cells and neutrophils in muscle injury immune microenvironment inhibits muscle stem‐cell mediated regeneration leading to fibrotic scar. In addition, neutrophils could release profibrotic cytokines, including IL‐17,^[^ ^103^ ^]^ IL‐6, and VEGF.^[^ ^82^ ^]^ Neutrophils‐produced ROS induces the activation and proliferation of hepatic stellate cells (HSCs); meanwhile, activated HSCs could secrete IL‐15 and GM‐CSF to inhibit the neutrophils apoptosis in liver fibrosis (Figure 3A).^[^ ^104^ ^]^

Interestingly, neutrophils participate in the transport of pre‐existing matrix across organs depending on the HSF‐integrin AM/B2‐kindlin3 cascade. Pharmacologic inhibition of this axis and chemokine receptor CXCR2 blocks neutrophils carrying fibrotic matrix to the wounds, a therapeutic beacon to alleviate scaring.^[^ ^105^ ^]^ NLRP3 activation in neutrophils induces liver fibrosis by recruiting inflammatory neutrophils, activating pro‐inflammatory signaling in infiltrating myeloid cells and reducing the number of Kupffer cells.^[^ ^106^ ^]^ In myocardial infarction (MI) mice, NLRP3 inflammasome activation in neutrophils via calcium‐sensing receptor promotes myocardial apoptosis and fibrosis.^[^ ^107^ ^]^ These results remind us that NLRP3 of neutrophils may be a potential target in fibrosis treatment.

Neutrophil elastase (NE) is a serine protease normally expressed in neutrophil primary granules and thought to be essential in degrading some ECM proteins including collagens I–IV, laminin, fibronectin, elastin, and entactin, which help neutrophils destroy the structure of the pathogen and subsequent lysis.^[^ ^108^ ^]^ Several studies show that NE promotes cardiac, lung, and liver fibrosis by promoting myofibroblast differentiation, TGFβ activation, inflammatory cell recruitment, EMT, and matrix production; particularly, inhibition or deletion of NE substantially reverses fibrosis in mice.^[^ ^109^, ^110^, ^111^ ^]^ Hypoxia in COPD not only augments NE release but also induces a destructive—“hypersecretory” neutrophil phenotype, resulting in endothelial damage and further tissue injury (Figure 3A).^[^ ^112^ ^]^

NETs play important roles both in anti‐infection and tissue damage. However, it is generally believed that the NETs are beneficial to host defense, but also have a predominately detrimental role in tissue damage and repair. In chronic thromboembolic pulmonary hypertension, the increase of NETs formation promoting monocyte differentiation to activate fibroblast leads to fibrotic thrombus remodeling.^[^ ^113^ ^]^ Another study shows that NETs activate fibrocytes and promote the differentiation of monocytes into fibrocytes, which migrate into the infarcted site to induce adverse remodeling during ST‐segment elevation myocardial infarction.^[^ ^114^ ^]^ Moreover, NETs formations induce EMT and fibrogenesis in benzyl butyl phthalate and COVID‐19‐induced lung injury.^[^ ^115^, ^116^ ^]^ Caspase‐11/GSDMD‐dependent NETs promote renal fibrosis in obstructive nephropathy via facilitating renal inflammation and MMT.^[^ ^61^ ^]^ Deletion of histidine decarboxylase in neutrophils, inhibiting histamine generation, aggregates cardiomyocyte death and cardiac fibrosis by enhanced neutrophil infiltration and NET formation.^[^ ^117^ ^]^ NET contents, including DNA, histones, and myeloperoxidase, trigger the differentiation of lung fibroblast into myofibroblast and endothelium damage.^[^ ^118^ ^]^ In interstitial lung diseases, NETs and their contents activate lung fibroblasts and the differentiation into myofibroblasts via the TLR9‐miR‐7‐Smad2 signaling pathway.^[^ ^119^ ^]^ Recently, NET‐associated proteins, integrin‐αvβ1 and MMP9, have been shown to activate latent TGFβ to promote EMT in cancer,^[^ ^120^ ^]^ indicating NETs probably activate TGFβ signaling to trigger fibrogenesis. Moreover, NETs decorated with tissue factor (TF) and interleukin‐17A (IL‐17A) can promote thrombin generation and pro‐fibrotic potential of fibroblasts to mediate end‐organ injury and fibrosis in systemic lupus erythematosus.^[^ ^121^ ^]^ Interestingly, DNA degrading enzyme DNase has been reported to mitigate NET‐mediated pathology in numerous inflammatory diseases in mice,^[^ ^122^ ^]^ which should be considered for fibrosis treatment options.

### 5.3 Anti‐Fibrotic Roles of Neutrophils

Although neutrophils are thought to play important roles in pro‐fibrosis mostly, several studies have shown anti‐fibrotic roles of neutrophils after tissue injury. MiR‐233 expressed in neutrophils silences NLRP3 in pro‐inflammatory macrophages and triggers the activation of restorative macrophage phenotype to release IL‐10, which inhibits HSCs activation and collagen deposition in early liver fibrosis.^[^ ^123^ ^]^ Moreover, neutrophils also express pro‐reparative proteases, such as MMPs. In liver fibrosis, neutrophils can produce MMP8 and MMP9 to degrade collagen fibrotic matrix.^[^ ^124^ ^]^ MMP9 and MMP2 released from neutrophils correlate with better outcomes in ventilator‐induced lung injury and adequate matrix deposition during repair.^[^ ^125^ ^]^ Neutrophils also participate in cardiac and liver repair by producing neutrophil gelatinase‐associated lipocalin (NGAL) and ROS to promote macrophage polarization toward a reparative phenotype with high capacity for engulfing apoptotic cells (Figure 3A).^[^ ^126^, ^127^ ^]^ Neutrophils could release HGF, a pleiotropic cytokine regulating the survival and proliferation of hepatocytes, to participate in liver regeneration rather than continuous fibrosis in alcoholic hepatitis.^[^ ^128^ ^]^ Continuous inflammation response leads to fibrosis; while, NETs and their components have been shown to scavenge proinflammation cytokines and chemokines;^[^ ^129^ ^]^ so that, neutrophils may alleviate fibrosis by accelerating inflammation resolution.

## 6 Basophils, Eosinophils, and Mast Cells

Except for neutrophils, granule‐containing leukocytes also include basophils, eosinophils, and mast cells. These three granulocytes have been linked to the host responses to pathogens and allergens. In addition to being potent effector cells in host defense, they still function as modulators of immune responses, as well as of tissue remodeling and repair, by releasing pre‐formed or newly synthesized effector molecules including granule proteins, biogenic amines, proteases, cytokines, chemokines, growth factors, and lipid mediators.^[^ ^130^, ^131^ ^]^ Although the in vivo functions of these cell types are not well understood due to rare numbers, short lifespan (basophils and eosinophils), and difficulty of isolation, their roles in fibrosis are gradually discovered.

Aberrant and persistent type 2 immunity results in the pathogenic fibrosis process, suggesting the crucial roles of basophils, eosinophils, and mast cells in fibrosis‐related diseases. Damaged epithelial cells secrete IL‐25, IL‐33, and TSLP upon injury; and then, these alarm signals trigger type 2 immunity by producing type 2 cytokines IL‐4, IL‐5, and IL‐33 by innate immune cells.^[^ ^132^ ^]^ Basophils, mast cells, and eosinophils‐released IL‐4 and IL‐13 promote T helper 2 cell development and myofibroblast activation.^[^ ^133^ ^]^ Interestingly, eosinophils‐derived IL‐4 and IL‐13 contribute to both tissue regeneration and progressive fibrosis. When undergoing temporary injury, eosinophils release these type 2 effector cytokines to augment the proliferation of epithelial and parenchymal cells, and production of extracellular matrix by fibroblasts, as well as inhibit DCs antigen presentation and T helper 2 cells activation to aid tissue repair. When type 2 cytokines‐mediated regeneration transforms to chronic or dysregulated, fibroblasts secrete excessive ECM in response to eosinophils‐released IL‐4 and IL‐13.^[^ ^133^ ^]^ In addition, basophils‐derived IL‐4 and IL‐13 play central roles in the healing process without cardiac remodeling by promoting reparative macrophage differentiation and inflammation resolution;^[^ ^134^ ^]^ while, basophils‐derived IL‐4 plays a crucial role in cardiac allograft fibrosis by promoting fibroblast activation, myofibroblasts expansion, and ECM deposition (Figure 3C).^[^ ^135^ ^]^ Considering the intricate roles of type 2 cytokines, especially IL‐4 and IL‐13, in regeneration and fibrosis processes, targeting type 2 cytokines may not yield the desired effectiveness.

Mast cells act as effective sensors and regulators of inflammatory response and the subsequent development of fibrosis. Activated mast cells can promote the proliferation, activation, and differentiation of fibroblasts by releasing TGFβ, histamine, and granule contents such as tryptase and chymase through degranulation.^[^ ^136^ ^]^ Mast cell‐produced proteases, chymase and tryptase, also contribute to cardiac fibrosis by triggering cardiomyocyte apoptosis.^[^ ^137^ ^]^ In addition, mast cell‐released renin prompts the production of Angiotensin II, which is highly arrhythmogenic and responsible for subsequent fibrosis.^[^ ^138^ ^]^ Interestingly, TNFα and proteases such as tryptase and chymase, released by mast cells, can activate MMPs to induce fibrillar collagen degradation.^[^ ^139^ ^]^ Thus, mast cell‐derived proteases have both detrimental and favorable effects on tissue remodeling, indicating the difficulty in targeting tryptase or chymase. Intercellular crosstalk can occur not only through soluble factors but also through cell contact. In skin fibrosis, keratinocytes secrete PAI‐1 to increase mast cell infiltration and upregulate ICAM1 expression on dermal fibroblasts, which leads to mast cell‐fibroblast heterotypic cell connections, resulting in sustained coactivation and final fibrogenesis.^[^ ^140^ ^]^ Therefore, in addition to blocking the release of cytokines and granule contents from mast cells, hindering the contact between mast cells and fibroblasts is another way to prevent fibrosis process. IL‐9, released by IL‐33‐expanded ILC2, has been shown to drive a positive feedback loop to enhance lung inflammation in cystic fibrosis through the mast cell‐ILC2‐Th9 pathway. ILC2‐produced IL‐9 induces mast cells to secret IL‐2, leading to CD25^+^ ILC‐2 expansion and further, to IL‐9 releasing to prompt ILC2 survival, type 2 cytokines production, Th9 cell activation, and profibrotic TGFβ production by mast cells (Figure 3C).^[^ ^141^ ^]^ Blocking IL‐9 or inhibiting mast cells may restrict the chronic inflammation and allergy process. During NAFLD progression to NASH, biliary‐secreted IGF‐1 enhances mast cell infiltration and activation, leading to severe biliary and liver damage, and microvesicular steatosis particularly in portal areas hepatocytes via miR‐144‐3p/ALDH1A3 signaling.^[^ ^142^ ^]^ Cell‐surface receptor KIT and its ligand, stem cell factor (SCF), have diversiform effects on mast cell development and function. SCF‐KIT interactions mediate mast cell differentiation, proliferation, maturation, survival, migration, chemotaxis, adhesion, degranulation, and even biological effector molecule release.^[^ ^143^ ^]^ Consequently, targeting the SCF‐KIT pathway and IGF‐1 provides us with new insights into regulating the various functions of mast cells during the fibrotic process.

Eosinophil accumulation in the vascular system and tissues is associated with numerous inflammatory and infectious diseases. As research on the functions of eosinophils deepens, their role in fibrosis is gradually attracting attention. IL‐4 and IL‐5‐mediated eosinophil infiltration has been shown associated with interstitial fibrosis, which is enhanced by eosinophil‐derived profibrotic TGFβ.^[^ ^144^ ^]^ Eosinophils‐derived TGFβ and IL‐1β prompt fibroblast activation, IL‐6 secretion, and ECM production, while fibroblast‐derived GM‐CSF induces eosinophil activation and prolonged survival.^[^ ^145^, ^146^ ^]^ Eosinophil‐released IFN‐γ, independent of lymphocytes, is also essential for eosinophils‐induced lung fibrosis.^[^ ^147^ ^]^ Amphiregulin, produced by ST2^high^ memory Th2 cells, reprograms the transcriptome of eosinophils toward an inflammatory phenotype to produce profibrotic osteopontin.^[^ ^148^ ^]^ However, eosinophil‐produced IL‐4 and cationic protein mEar1 can inhibit hypoxia or H_2_O_2_‐induced cardiomyocyte apoptosis, fibroblast activation and ECM production, and inflammatory cells adhesion post‐MI, indicating the anti‐fibrotic roles of eosinophils (Figure 3C).^[^ ^149^ ^]^

## 7 Innate Lymphoid Cells

ILCs are a previously unappreciated cell type of the innate immune system lacking antigen specific receptors expressed on T cells and B cells. The ILC family contains ILC1, ILC2, ILC3, and NK cells, which are considered as the innate counterparts of the T lymphocytes. In the past decades, research on the biology of ILCs has expanded beyond their role as a bridge between innate and adaptive immunity, involved in regulating immune responses to inflammation or infection, to include tissue remodeling, metabolic homeostasis, cancer, and the regulation of the neural system.^[^ ^150^, ^151^ ^]^ In addition, numerous studies of fibrosis‐related diseases have identified conspicuous alterations in ILC responses, indicating the potential roles of ILCs in fibrosis process.

**Figure 4:**
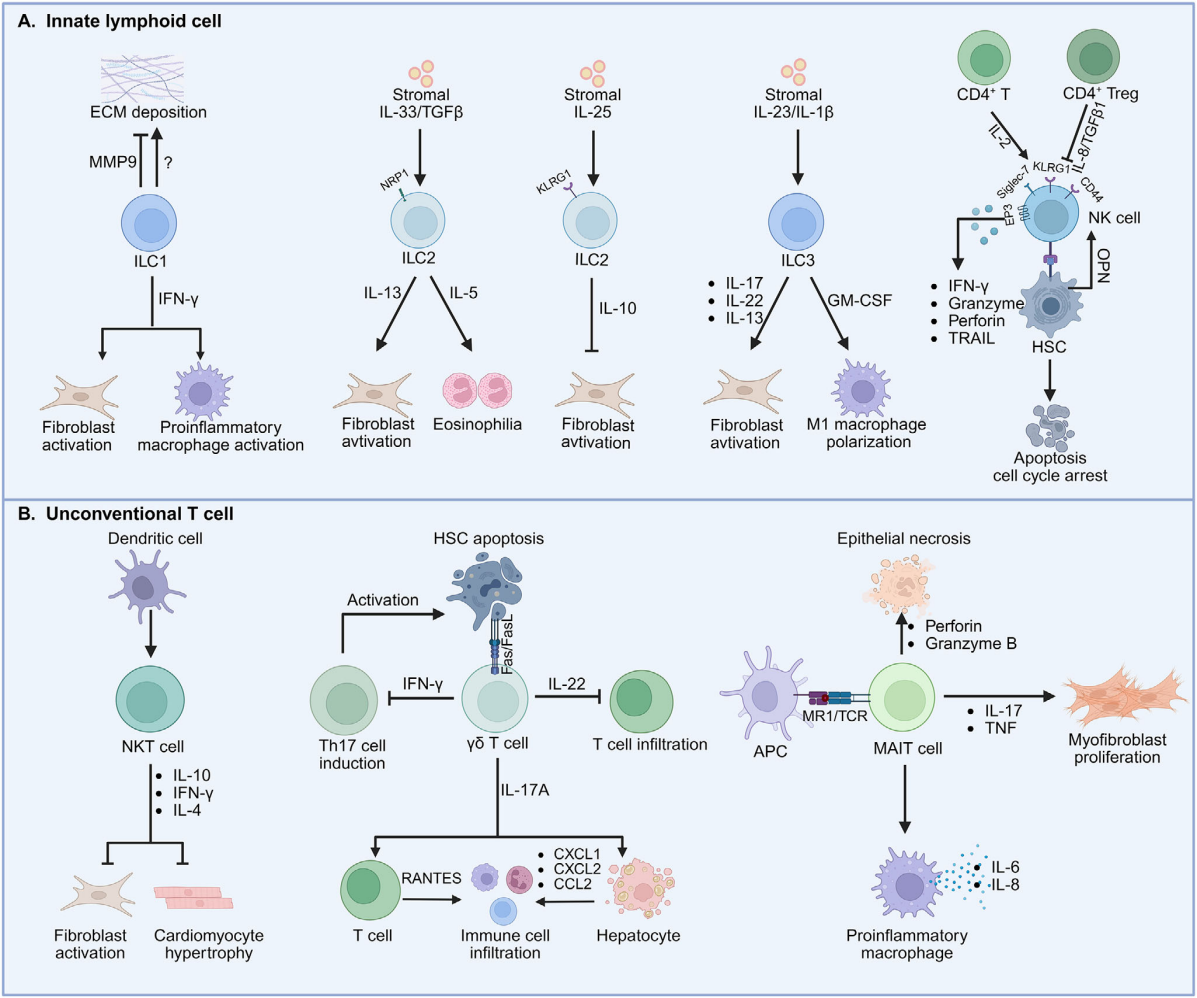
A) Innate lymphoid cell: friends or foes? Upon tissue injury, ILC1 not only releases MMP9 to inhibit ECM deposition but also secretes IFN‐γ to induce the activation of pro‐inflammatory macrophages and fibroblasts. Stromal IL‐33 or TGF can induce ILC2 to adopt a pro‐fibrotic phenotype, releasing IL‐12 to activate fibroblasts and secreting IL‐5 to trigger eosinophilia. On the contrary, Stromal IL‐25 can induce ILC2 to adopt an anti‐fibrotic phenotype, releasing IL‐10 to inhibit fibroblast activation. For ILC3, matrix‐derived IL‐23 and IL‐1β induce the production of IL‐17, IL‐22, and IL‐13, which activate fibroblasts, as well as GM‐CSF to promote the polarization of M1 macrophages. In addition to releasing cytoplasmic granules containing TRAIL, perforin, and granzyme, the killing process of NK cells against HSCs depends on NKG2D, NKp46, Siglec‐7, and E‐prostanoid 3 receptors expressed by NK cells and NCR1 ligands expressed by HSCs. B) Unconventional T cells as pleiotropic orchestrators in fibrotic pathogenesis. After CD1‐expressing DCs activate NKT cells, NKT cells can produce IL‐10, IL‐4, and other factors to promote cardiomyocyte hypertrophy and fibroblast activation to contribute to fibrosis. When damaged, γδ T cells‐secreted proinflammatory cytokine IL‐17A directly activates CD3^+^ T cells to produce chemokine RANTES; and then, recruits macrophages/T cells to promote inflammatory response and fibrosis. Moreover, IL‐17A can induce hepatocytes to produce chemokines (CCL1/CXCL1/CXCL2), which facilitate proinflammatory cell infiltration. γδ T cells also perform pro‐repair functions by producing IL‐22 and IFN‐γ to inhibit T cell infiltration and differentiation or inducing HSC apoptosis via the Fas‐FasL pathway. MAIT cells can produce perforin and granzyme B to trigger epithelial cell necrosis. Further, MAIT cell regulates proinflammatory macrophage phenotypic transition, which releases IL‐6 and IL‐8 to promote chronic inflammation. IL‐17 and TNF secreted from MAIT cells contribute to fibrosis by triggering myofibroblast proliferation. Created with BioRender https://www.biorender.com/.

Considering that both ILC1 and NK cells can produce IFN‐γ as their principal effector cytokine and rely on transcription factor T‐bet for this function, these two cells are generally classified as group 1 ILCs. Despite their differences in differentiation path and function, they are difficult to distinguish especially when encountering infection and inflammatory conditions.^[^ ^152^ ^]^ Therefore, exploring the role of ILC1 and NK cells in chronic inflammatory diseases is challenging, and relevant research on ILC1 is still lacking due to the phenotypic characterization being problematic. ILC1 derived from patients with inflammatory bowel disease can release TGFβ1 to drive the expansion of CD44v6^+^ epithelial crypts. In addition, ILC1 also triggers the fibronectin 1 deposition and MMP9 release to mediate ECM degradation, indicating that ILC1 regulates matrix softening and stiffening.^[^ ^153^ ^]^ ILC1 has been reported to promote proinflammatory CD11c^+^ macrophage activation, TGFβ signaling activation, and adipose tissue fibrosis via IFN‐γ production. Using IL‐12 neutralizing antibodies to restrain ILC1 proliferation and accumulation attenuates fibrosis and improves glycemic tolerance,^[^ ^154^ ^]^ providing a treatment strategy for fibrosis associated with metabolic diseases. As a primary effector of innate immune response, NK cells demonstrate strong cytolytic properties; so, their anti‐fibrotic effect is attributed to directly killing activated hepatic stellate cells.^[^ ^155^ ^]^ This killing process relies on the NKG2D, NKp46, Siglec‐7, and E‐prostanoid 3 receptor expressed by NK cells, as well as the NCR1 ligand expressed by HSCs,^[^ ^156^, ^157^, ^158^ ^]^ suggesting manipulation of these receptors; and corresponding ligand activity may help in the treatment of fibrosis to some extent. Interestingly, killer cell lectin‐like receptor subfamily G member 1 (KLRG1) is expressed by terminally differentiated and mature NK cells. Compared to KLRG1^−^ NK cells, KLRG1^+^ NK cells have stronger cytolytic activity and IFN‐γ production to induce HSC apoptosis; while, HSC‐derived osteopontin promotes KLRG1^+^ NK cell activation.^[^ ^159^ ^]^ Metabolic changes in the microenvironment are also involved in functional transformation of cells. The increased uptake of glutamate by activated HSCs in the microenvironment results in decreased levels of glutamate, which hampers the activation of mGluR5 in NK cells, leading to more severe fibrosis. Activation of mGluR5 in NK cells increases the anti‐fibrosis genes expression, IFN‐γ production, and cytotoxicity against HSCs, suggesting novel roles of glutamate metabolism and mGluR5 activation in fibrosis.^[^ ^160^ ^]^ However, CD4^+^ Tregs can interfere with the anti‐fibrotic effects of NK cells through direct cell‐contact‐dependent inhibition and release of soluble factors such as IL‐8 and/or TGF‐β1 which downregulate essential ligands responsible for NK cell activation on HSCs;^[^ ^161^ ^]^ while, CD4^+^ T cells supernatants enhance the anti‐fibrotic activity of NK cells, probably due to CD4^+^ T cell‐released IL‐2 (**Figure** **4**A).^[^ ^162^ ^]^ Hence, in addition to the communication and interaction between NK cells and HSCs, the immune microenvironment plays an important role in regulating the activation and anti‐fibrosis function of NK cells.

ILC2 is the most extensively studied cell type among the innate lymphoid cells in relation to fibrosis. IL‐33, released by endothelial cells or epithelial cells in the tissue microenvironment, is responsible for ILC2 infiltration, activation, and expansion. ILC2 has been shown to be involved in hepatic,^[^ ^163^ ^]^ skin,^[^ ^164^ ^]^ and endometrium^[^ ^165^ ^]^ fibrosis, respectively. In diseased muscle, stromal progenitors‐produced IL‐33 induces ILC2 expansion, which drives muscle eosinophilia via IL‐5 secretion, resulting in further fibrosis gene transcription. Thus, IL33‐ILC2 may be a pro‐fibrotic axis and a target of great potential. TGFβ‐Nrp1 signaling enhances ILC2 function through upregulating IL‐33 receptor ST2 expression; while, genetic ablation or pharmacological inhibition of Nrp1 can inhibit IL‐5 and IL‐13 production by ILC2 and alleviate pulmonary fibrosis.^[^ ^166^ ^]^ Phospholipid scramblase‐1 (PLSCR1), a type II transmembrane protein, regulates ILC2 activation and innate type 2 responses via CRTH2‐dependent manners.^[^ ^167^ ^]^ Moreover, genetic deletion of Regnase‐1 in ILC2 contributes to proliferation, activation of ILC2, and profibrotic gene expression, implying Regnase‐1 performs as a critical regulator of the profibrotic function of ILC2.^[^ ^168^ ^]^ Considering that Nrp1, Regnase‐1, and PLSCR1 play essential roles in the pathogenesis of ILC2 responses, manipulating them to regulate fibrosis development represents a new therapeutic direction. Functional and phenotypic heterogeneity of ILC2 have not been well characterized. Interestingly, TGFβ in the environment supports the expansion of KLRG1^−^ ILC2 (which responds to IL‐33), instead of KLRG1^+^ ILC2 (which responds to IL‐25), leading to diminished IL‐10 production by ILC2 and increased myofibroblasts differentiation.^[^ ^164^ ^]^ Further, an IL‐5^+^ ILC2 niche sustained by IL‐33^high^ adventitial fibroblasts restrains IL‐17‐mediated liver fibrosis.^[^ ^169^ ^]^ These results suggest the profibrotic role of KLRG1^−^ ILC2 and the anti‐fibrotic role of IL‐5^+^ ILC2; while, further exploration is needed regarding the heterogeneity of ILC2. IL‐33‐activated ILC2 cells release IL‐9 to activate mast cells capable of producing pro‐fibrotic TGFβ, as well as activate Th9 cells, thereby amplifying the inflammatory cycle and promoting fibrosis.^[^ ^141^ ^]^ Targeting ILC2‐produced IL‐9 and type 2 cytokines may alleviate ILC2‐induced fibrosis (Figure 4A).

ILC3 mirrors CD4^+^ T helper (Th) 17 cells, in terms of function, and combats extracellular microbes. ILC3 participates in type 3 immunity through producing diverse cytokines, including IL‐22, IL‐17, GM‐CSF, and lymphotoxin.^[^ ^150^ ^]^ ILC3‐derived IL‐17A, IL‐13, and IL‐22 contribute to renal^[^ ^170^ ^]^ and liver^[^ ^171^ ^]^ fibrosis. In addition, released IL‐22 can inhibit anti‐fibrotic cytokine IFN‐γ production by other immune cells in the microenvironment. The inhibition of cytokine production derived from ILC3, the depletion of ILC3, and the genetic deletion of *Rora*, which regulates the production of these cytokines, can all lead to decreased fibrosis.^[^ ^172^, ^173^ ^]^ GM‐CSF, produced by ILC3, regulates macrophage polarization toward pro‐inflammatory M1 macrophages, instead of pro‐repair M2 phenotype (Figure 4A).^[^ ^174^ ^]^ Consequently, the role of ILCs in fibrotic diseases is still lacking, probably due to the insufficient understanding of ILCs. Targeting cytokines produced by ILC3 is a potential means to treat fibrosis for the moment.

## 8 Unconventional T Cells

Natural killer T (NKT) cells, mucosal‐associated invariant T (MAIT) cells, and γδ‐T cells are the three best‐studied unconventional T cell key subsets. Unlike conventional T cells, unconventional T cells are restricted to monomorphic major histocompatibility complex (MHC) and T cell receptor (TCR) repertoire analogously to an innate immune receptor. Interestingly, they can recognize non‐peptide antigens such as lipids, metabolites, and phosphoantigens dependently or independently of MHC‐like antigen‐presenting molecules.^[^ ^175^ ^]^ As the antigen‐mediated activation of these unconventional T cells is not pre‐requisite, these innate‐like T cells accumulate earlier and faster than conventional T cells and are accompanied by rapid cytokine responses. Therefore, unconventional T cells, considered as a bridge between innate and adaptive immunity, are indispensable subsets of T cells and valued regulators of tissue homeostasis, inflammation, repair, pathogen infection, and cancer.^[^ ^176^, ^177^ ^]^ While there is some overlap in their roles, the differences in their localization in tissues and their ability to recognize different MHC molecules imply that they have unique functions in tissue damage response. Figuring out their roles in fibrogenesis will facilitate further understanding of the pathology of fibrosis‐related diseases and the search for suitable therapeutic targets.

Natural killer T (NKT) cells are a special T lymphocyte subset expressing both NK receptors and invariant TCRs. Based on differences in TCR characteristics, NKT cells are divided into two categories: type I NKT cells (iNKT) using a semi‐invariant TCR and type II NKT cells using a more diverse TCR repertoire. In the thymus, iNKT cells differentiate into three subsets, NKT1, NKT2, and NKT17, which are analogous to Th1, Th2, and Th17 CD4^+^ T cells. After epigenetic and transcriptome remodeling, iNKT cells egress from the thymus and colonize the liver, lung, spleen, and other tissues throughout the body.^[^ ^178^ ^]^ These non‐circulating and tissue‐resident iNKT cells are heterogeneous and, once activated, produce a variety of cytokines to regulate tissue homeostasis and damage repair.^[^ ^178^, ^179^ ^]^ A previous study has shown that iNKT cells are increased in steatohepatitis compared to steatosis,^[^ ^180^ ^]^ indicating the crucial role of iNKT cells in liver fibrosis. In the early stage of steatohepatitis, the recruited iNKT cells undergo transcriptome‐level changes and aggregate into small clusters, which promote lipid phagocytosis and clearance by dynamically interacting with Kupffer cells.^[^ ^181^ ^]^ Thus, identifying the lipid‐sensitive iNKT cell subsets and unraveling how these cells exert their phagocytosis and clearance functions would be beneficial in preventing the progression of liver steatosis to fibrosis. However, iNKT cell‐produced IL‐4 could induce HSCs membrane protein GARP expression to promote liver fibrosis through TGFβ activation.^[^ ^182^ ^]^ These findings suggest a shift in the pro‐fibrotic and anti‐fibrotic roles of iNKT cells across distinct stages of liver fibrosis, which may account for their cellular heterogeneity. In addition to liver tissues, the functional studies of NKT cells in other tissue damage repair are still lacking. Activation of NKT cells, regulated by CD1d‐expressing DCs, induces IL‐10 production to inhibit cardiomyocyte hypertrophy and fibroblast differentiation through the activation of STAT3 and inhibition of TGFβ and NF‐kB pathways.^[^ ^183^ ^]^ In lung fibrosis with pulmonary hypertension, the deficiency of NKT cells results in a lower concentration of IFN‐γ, which increases collagen deposition by inhibiting STAT1 signaling in pulmonary arterial smooth muscle cells.^[^ ^184^ ^]^ Therefore, the administration of NKT cell‐produced cytokines, such as IL‐10 and IFN‐γ, or pharmacological activation of NKT cells might improve tissue remodeling (Figure 4B).

MAIT cells can be activated via combined inflammatory cytokines or TCR ligands presented by MR1. The mode of activation determines the function of MAIT cells. TCR signals‐triggered MAIT cells activation generally promotes the secretion of effector molecules regulating tissue homeostasis and wound repair; while, inflammatory cytokines‐triggered activation generally promotes the pro‐inflammatory effector molecules.^[^ ^185^, ^186^ ^]^ During cirrhosis, MAIT cells exhibit functional alterations, with an accumulation of surrounding fibrogenic cells in fibrotic zones. Mechanistically, these MR1‐dependent activated MAIT cells, producing IL‐17 and TNF, function as a profibrogenic immune cell population through contact with myofibroblasts directly or indirectly to promote myofibroblasts proliferation. Moreover, activated MAIT cells stimulate the production of proinflammatory cytokines IL‐6 and IL‐8 by monocyte‐derived macrophages.^[^ ^187^ ^]^ The interaction of MAIT cell‐monocyte/macrophage also reprograms macrophage signature from restorative Ly6C^low^ to a pro‐inflammatory Ly6C^high^ macrophage phenotype.^[^ ^188^ ^]^ In chronic kidney disease, hypoxic proximal tubular epithelial cells and inflammatory environment including IL‐18, IL‐12, and IL‐15 activate the tissue‐resident MAIT cells. Then, activated MAIT cells upregulate the expression of CD69 and the production of cytotoxic molecules, perforin, and granzyme B, which induce PTEC necrosis in turn (Figure 4B).^[^ ^189^ ^]^

Similar to MAIT cells and NKT cells, γδ T cells are also enriched in many peripheral tissues, such as the liver, skin, lung, and intestines. A large fraction of γδ T cells regulates tissue repair and immune response by producing IFN‐γ and IL‐17A.^[^ ^190^ ^]^ Lipids accumulated in hepatocytes cause γδ T cell activation through the immune receptor NKG2D and promote the production of IL‐17A. IL‐17A triggers hepatocytes to release chemokines such as CXCL1/2 and CCL2, which result in proinflammatory cell infiltration and liver fibrosis.^[^ ^191^ ^]^ Both in UUO‐treated mice and tubulointerstitial fibrosis patients, γδ T cells‐secreted proinflammatory cytokine IL‐17A is increased, which directly activates CD3^+^ T cells to produce chemokine RANTES; and then, recruits macrophages/T cells to promote inflammatory response and fibrosis.^[^ ^192^, ^193^ ^]^ In addition, hepatic γδ T cells can protect against liver fibrosis by cytotoxicity against activated HSCs and producing IFN‐γ to suppress the profibrotic Th17 cell differentiation.^[^ ^194^ ^]^ IL‐22 secreted by γδ T cells represses lung fibrosis by inhibiting the recruitment of CD4^+^ T cells (Figure 4B).^[^ ^195^ ^]^ Thus, the administration of recombinant anti‐fibrotic IL‐22/IFN‐γ or blocking of pro‐fibrotic IL‐17A may be a potential strategy to improve fibrosis.

## 9 T Cells

T cell is a major type of lymphocyte in the immune system. Increasing studies reveal that the infiltration of T cells, particularly CD4^+^ T cells, is associated with tissue fibrosis,^[^ ^196^, ^197^, ^198^ ^]^ in the liver, kidney, and pancreas. Th2 cell‐produced IL‐4 and IL‐13 promote macrophage activation, which induces the activation of fibroblasts in turn. However, CD25^+^FOXP3^+^ Treg cells can downregulate the Th2‐mediated type 2 immune response to repress pancreatic fibrosis.^[^ ^198^ ^]^ Moreover, the dysregulated Th17/Treg ratio contributes to increased type 3 cytokines IL‐17 and IL‐22 in the immune microenvironment, which results in HSCs activation through intensive TGFβ response.^[^ ^103^ ^]^ Of note, Th1 cells mediate fibroblast proliferation and activation through the IFN‐γ/STAT1 pathway; while, Treg blocks aberrant Th1 immune response.^[^ ^199^, ^200^ ^]^ These results indicate the balance of three types of T helper cell responses and Treg response might be a suitable target. Considering tissue‐resident Treg exhibits complex and flexible phenotypic and functional transformations depending on the inflammatory environment,^[^ ^197^ ^]^ the underappreciated plasticity of Treg cells in inter‐organ and intra‐organ requires further investigation to more effective target Tregs. In SSc, CD8^+^ T cells and CCR7^−^ CD4^+^ memory T cells can produce IL‐13 to promote the transition of fibroblasts to myofibroblasts and ECM production.^[^ ^82^ ^]^ Moreover, the increased myocardial infiltration of T lymphocytes and macrophages is an important pathological feature of immune checkpoint inhibitor (ICI) myocarditis. Recent studies show that IFN‐γ‐producing CD8^+^ T cells induce the expansion of a monocyte‐derived *Cxcl9* ^+^ *Cxcl10* ^+^ macrophage subset to potentiate myocardial inflammation and remodeling; while, blocking IFN‐γ signaling can alleviate ICI myocarditis.^[^ ^201^, ^202^ ^]^ In autoimmune myocarditis, Th17 cell‐derived IL‐17A is dispensable for the development of myocarditis. However, IL‐17A deficiency has been shown to prevent post‐myocarditis remodeling and progression to dilated cardiomyopathy by inhibiting the production of profibrotic molecules (e.g., IL‐1β, IL‐6, TNFα, and TGFβ1) and recruitment of myeloid populations.^[^ ^203^ ^]^ These studies highlight the pivotal role of T cells in autoimmune fibrosis and suggest that targeting cytokines including IL‐17A, IL‐13, and IFN‐γ may represent a promising strategy for therapeutic intervention. In addition, single‐cell transcriptome and FACS analysis identify a unique CD8^+^ tissue‐resident memory T (CD8^+^ Trm) cell subset maintained by tissue IL‐15 in NASH resolution liver. The CD8^+^ Trm cells attract HSCs in a CCR5‐dependent manner and exert cytotoxic effects to induce FasL‐Fas‐mediated apoptosis of activated HSCs.^[^ ^204^ ^]^ Although researchers have attempted to explore the heterogeneity of T cell function in the fibrosis process at the single‐cell transcriptome level, the underlying mechanisms, especially how they interact with other cells in the tissue microenvironment, need to be further addressed.

## 10 B Cells

Increasing studies show that B cells are accumulating adjacent to the fibrosis niche in IPF and hepatitis patients.^[^ ^205^, ^206^ ^]^ Surprisingly, B cell activation during NASH involves both MyD88‐mediated innate signaling and BCR‐mediated adaptive signaling, targeting the activation of B cells such as MyD88 ameliorating liver fibrosis.^[^ ^207^ ^]^ In blood samples of SSc patients, naïve and activated memory B cells and B cell activating factor (BAFF) levels are positively correlated with disease severity.^[^ ^208^ ^]^ Cholangiocytes‐released CXCL12, elevated in chronic liver diseases, contributes to profibrotic proinflammatory CXCR4‐expressing B cell infiltration.^[^ ^205^ ^]^ Infiltrated B cells perform as a profibrotic macrophage differentiation inducer depending on secreted IL‐6 and CD11a‐CD22‐mediated direct contact.^[^ ^209^ ^]^ Moreover, B cells promote fibrosis progression through limiting HSCs senescence and triggering the Th1 immune response.^[^ ^207^, ^210^ ^]^ However, a previous study shows that IL‐10‐producing regulatory B cells (Bregs) alleviate tissue remodeling by repressing CCR2 expression in monocytes to inhibit proinflammatory monocyte recruitment and mobilization,^[^ ^211^ ^]^ indicating the beneficial effect of Bregs in inflammation resolution. Depleting BAFF exhibits potential for SSc treatment via altering the balance of anti‐fibrotic IL‐10‐producing Bregs and pro‐fibrotic IL‐6‐producing effector B cells.^[^ ^208^ ^]^ In addition, CD1d‐lipid presentation by CD1d^+^T2‐MZP Bregs mediates the differentiation of suppressive iNKT to downregulate Th1 and Th17 immune responses, partially depending on the IFN‐γ production.^[^ ^212^ ^]^ Thus, Bregs‐mediated immune tolerance and inflammation inhibition provide new therapeutic avenues for chronic injury‐induced scar formation.

## 11 Immune Effector‐Driven Profibrotic Cell Reprogramming

**Figure 5:**
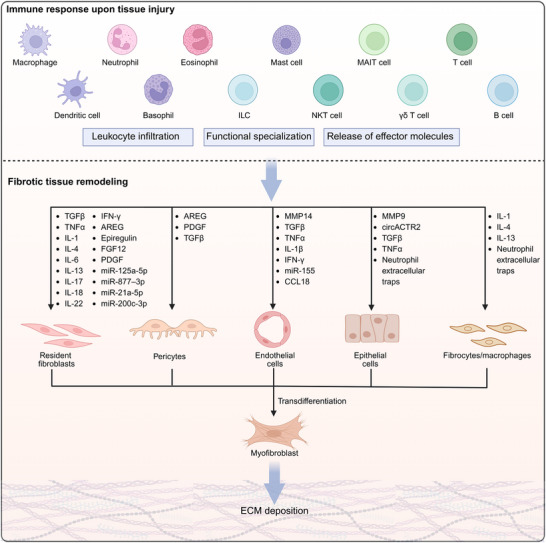
Immune microenvironment alteration‐driven regulation of ECM‐producing cells during tissue fibrosis. Upon tissue injury, site‐specific stimuli trigger leukocyte infiltration and proliferation of tissue‐resident immune cells. During this process, in response to dynamic microenvironmental alterations, immune cells undergo phenotypic and functional transformations and release diverse pro‐fibrotic effector molecules. These mediators contribute to scar formation by activating fibroblasts and inducing the transdifferentiation of epithelial cells, pericytes, endothelial cells, and circulating CD45‐positive hematopoietic lineage cells (including fibrocytes and macrophages) into ECM‐producing myofibroblasts. Created with BioRender https://www.biorender.com/.

The transient activation of myofibroblasts generates appropriate ECM to promote tissue repair; while, the continuous activation leads to excessive production of ECM, thereby causing pathological fibrosis. Given that the majority of myofibroblast precursors originate from mesenchymal cells, previous investigations have predominantly focused on elucidating how fibroblasts respond to immune microenvironmental stimuli and acquire a pro‐fibrotic phenotype. Immune cell‐secreted miRNAs and cytokines such as TGFβ, TNFα, IFNγ, IL‐4, IL‐18, IL‐22, epidermal regulators, FGF12, miR‐21a‐5p, and miR‐877‐3p stimulate fibroblast activation and drive myofibroblast differentiation.^[^ ^37^, ^38^, ^43^, ^171^, ^199^, ^213^ ^]^ Beyond fibroblasts, emerging evidence indicates that epithelial cells, endothelial cells, pericytes, and circulating CD45‐positive cells of the haematopoietic lineage (including fibrocytes and macrophages) can transdifferentiate into myofibroblasts under specific microenvironmental cues.^[^ ^214^ ^]^ Lamina propria mononuclear cell‐produced TGFβ, IL‐1β, and TNFα contribute to IBD‐associated fibrosis through inducing the process of EndoMT.^[^ ^215^ ^]^ In prolonged COVID‐19, local accumulation of CCR7^+^ T cells and CCL18^+^ heme‐scavenging macrophages drive pathological upregulation of chemokines CCL21 and CCL18, which are mechanistically linked to EndoMT.^[^ ^216^ ^]^ Moreover, AREG, PDGF, and TGFβ regulate pericyte to myofibroblast transition.^[^ ^46^, ^217^ ^]^ Macrophage‐derived MMPs, especially MMP9, have been shown to contribute to renal fibrosis by promoting EMT.^[^ ^218^ ^]^ T cell‐derived TGFβ and TNFα are key mediators of EMT in fibrosis.^[^ ^219^, ^220^ ^]^ Interestingly, both CD4^+^ Th2 cells and NKT cells can produce the pro‐fibrotic cytokine IL‐4 to regulate the differentiation of bone marrow‐derived fibroblast progenitor cells (**Figure** **5**).^[^ ^221^, ^222^ ^]^ The findings suggest that elucidating the mechanisms through which extracellular matrix‐producing cells acquire fibrotic phenotypes under the influence of immune microenvironmental alterations will facilitate the identification of effective therapeutic targets to ameliorate or reverse fibrosis.

## 12 Clinical Progress and Translational Implications

So far, the U.S. FDA has approved several mechanistically distinct antifibrotic agents for organ‐specific fibrotic diseases, each with defined therapeutic benefits and limitations. Pirfenidone (TGF‐β inhibitor) and nintedanib (tri‐tyrosine kinase inhibitor) have been approved for IPF. The treatment of pirfenidone and nintedanib can slow down the decline of lung function and has a significant statistical effect on the decline of forced vital capacity.^[^ ^223^ ^]^ Further, both drugs can reduce the hospitalization rate and related mortality rate of respiratory diseases. However, their use has shown problems such as low efficacy, non‐specific organ targeting, significant adverse reactions, and high treatment costs. Among them, pirfenidone has gastrointestinal toxicity, liver toxicity, and photosensitivity; while, nintedanib has the risk of diarrhea and bleeding.^[^ ^224^, ^225^ ^]^ Resmetirom, a thyroid hormone receptor‐β agonist for NASH‐related fibrosis, demonstrates modest histological improvement (25.9% fibrosis regression vs 14.2% placebo) but lacks metabolic benefits and raises concerns regarding unvalidated long‐term outcomes.^[^ ^226^ ^]^ Momelotinib, targeting JAK/ACVR1 in myelofibrosis, and a 7‐year follow‐up of patient data showed clinical improvement occurred in 57% of patients, including 44% anemia and 43% spleen response, coupled with hematologic toxicities (thrombocytopenia, anemia) and neuropathy.^[^ ^227^, ^228^ ^]^ Collectively, existing therapies have modestly improved fibrosis management; while, their clinical application is limited by significant drawbacks. This underscores the critical need for innovative therapeutic strategies that target underlying pathophysiological mechanisms with enhanced precision.

### 12.1 Antifibrotic Strategies Targeting Immune Microenvironment

Previous antifibrotic drugs targeting the immune microenvironment in clinical trials are mostly focused on immune cell‐derived cytokines, exosomes, and granule‐associated enzymes. These targeted profibrotic cytokines from immune cells include TNFα, IL‐4, IL‐13, IL‐6, IL‐1, TGFβ, FGF, IFN‐γ, and IL‐1α.^[^ ^45^, ^229^ ^]^ Although most of these drugs have entered clinical phases II and III, only Pirfenidone improved lung function and slowed down the progression of pulmonary fibrosis in IPF patients. The intricate cellular crosstalk characteristic of fibrosis, coupled with the fact that the origin of these cytokines is not singular, rendered it challenging to specifically target profibrotic cytokines within the immune microenvironment. Further, many inflammatory mediators are involved in both the initiation and resolution of fibrosis. We need to capitalize on their beneficial aspects and counteract the detrimental ones as this approach will facilitate our objective of promoting regeneration. Therapeutics that target neutrophil elastase,^[^ ^108^, ^148^, ^230^, ^231^ ^]^ exosomes containing microRNA (miR21),^[^ ^232^ ^]^ and target immune cell infiltration through chemokine CCL2^[^ ^4^ ^]^ and chemokine receptor CCR2^[^ ^233^ ^]^ are under clinical evaluation (**Table** **1**).

**Table 1:** Clinical trial drugs targeting the immune microenvironment.

Category	Name	Target	Class	Disease	Phase	NCT	Outcome	Status
Inflammatory cytokines	Etanercept	TNFα	Recombinant protein	IPF	I	NCT00063869	Well tolerated; decreased in rate of disease progression	Completed
–	Lebrikizumab	IL‐13	Monoclonal antibody	IPF	II	NCT01872689	Well tolerated, with a favorable safety profile	Completed
–	Tralokinumab	IL‐13	Monoclonal antibody	IPF	II	NCT02036580	Acceptable safety and tolerability; not reduce disease progression	Completed
–	Romilkimab	IL‐13	Monoclonal antibody	dcSSc	II	NCT02921971	Improved in modified Rodnan skin score	Recruiting
–	QAX576	IL‐13	Monoclonal antibody	IPF	II	NCT01266135	Unknown	Discontinued
–	Tocilizumab	IL‐6	Monoclonal antibody	dcSSc	III	NCT02453256	The primary skin fibrosis endpoint was not met	Recruiting
–	Interferon‐gamma	IFN‐γ	Aerosol interferon‐gamma	IPF	I	NCT00563212	Unknown	Completed
–	Interferon‐gamma‐1b	IFN‐γ‐1b	Interferon‐gamma‐1b	Cystic fibrosis	II	NCT00043342	Unknown	Completed
–	Bermekimab (MABp1)	IL‐1α	Monoclonal antibody	SSc	II	NCT04045743	Unknown	Completed
Cluster of differentiation	Foralumab	CD3	Monoclonal antibody	NASH	II	NCT03291249	Unknown	Withdrawn
–	Tagraxofusp	CD123	Fusion protein	Myelofibrosis	II	NCT02268253	Unknown	Recruiting
Growth factors	Pirfenidone	TGFβ	Pyridone derivatives	IPF	Marketed	NCT00662038	Efficacious, well‐tolerated	Completed
–	Hydronidone	TGFβ	Pyridine derivatives	Liver fibrosis	III	NCT05115942	Unknown	Recruiting
–	TRK‐250	TGFβ	siRNA‐based oligonucleotide	IPF	I	NCT03727802	Safe and well‐tolerated	Completed
–	Luspatercept	TGFβ	Fusion protein	Myelofibrosis	III	NCT04717414	Unknown	Recruiting
–	Sotatercept	TGFβ	Fusion protein	Myelofibrosis	II	NCT01712308	Well‐tolerated	Completed
								
–	AVID200	TGFβ	Fusion protein	Myelofibrosis	I	NCT03895112	Well‐tolerated, rational, therapeutic agent for patients with myelofibrosis	Completed
Intracellular enzymes	Lonodelestat	Neutrophil elastase	Peptide inhibitor	Cystic fibrosis	II	NCT03748199	Safe and well‐tolerated	Completed
–	CHF 6333	Neutrophil elastase	Small molecule	Cystic fibrosis	I	NCT04010799	Unknown	Completed
miRNA	RG‐012	miR21	Antisense oligonucleotide	Alport syndrome	I	NCT03373786	Unknown	Completed
–	Remlarsen (MRG‐201)	miR‐29	Antisense oligonucleotide	Keloid	II	NCT03601052	Unknown	Completed
Chemokine	CNTO‐888	CCL2	Monoclonal antibody	IPF	II	NCT00786201	No effect on disease progression, DLCO, infection rates, or mortality	Terminated
Chemokine receptor	CCX140B	CCR2	Small molecule	DKD	I	NCT01447147	Decrease in albuminuria	Completed
–	Cenicriviroc	CCR2/CCR5	Small molecule	NASH	III	NCT03028740	Safe and well tolerated but has no effects	Terminated
–	Sirolimus	CXCR4	Small molecule	IPF	NA	NCT01462006	Acceptable safety profile	Completed

IPF, idiopathic pulmonary fibrosis; dcSSc, diffuse cutaneous systemic sclerosis; SSc, systemic sclerosis; DKD, diabetic kidney disease; NASH, nonalcoholic steatohepatitis; DLCO, diffusing capacity of the lungs for carbon monoxide.

### 12.2 Chimeric Antigen Receptor (CAR) Cell Therapy

Engineered chimeric antigen receptor (CAR) T cells have previously been produced for recognizing specific antigens on cancer cells and have made dramatic progress in cancer therapy. Researchers have considered whether activated fibroblasts, as the primary culprits in fibrosis, could also be specifically recognized and eliminated by CAR‐T cells. Fibroblast‐activation peptide (FAP), only detectable in heart‐activated fibroblasts, was selected to be targeted. In vivo, mice with FAP CAR‐T cells treatment after exposure to AngII/PE stimulation significantly repressed cardiac fibrosis compared with control mice.^[^ ^234^ ^]^ Senescent cells generally cause an inflammatory milieu, which ultimately causes tissue damage and fibrogenesis. Thus, eliminating senescent cells by CAR‐T cells may alleviate chronic impairment. Engineered CAR‐T cells that target urokinase‐type plasminogen activator receptor (uPAR), which is expressed on fibrogenic, senescent hepatic myofibroblasts, also ameliorate liver fibrosis in vivo.^[^ ^235^ ^]^ Interestingly, engineered bone marrow‐derived macrophages with a CAR to direct their phagocytic activity against uPAR‐expressing hepatic stellate cells or FAP‐expressing cardiac fibroblasts both demonstrated remarkable antifibrotic efficacy in mice.^[^ ^236^, ^237^ ^]^ The therapeutic application of CAR in fibrotic diseases is currently under active development and investigation. Future research may focus on the development of CAR‐NK cells, CAR‐γδ T cells, and CAR‐Treg cells for therapeutic intervention.

Even though recent studies have unveiled the potential of CAR‐cell therapy in treating heart and liver fibrosis, several critical limitations and considerations remain to be addressed. Conventional CAR cell therapies required the separation of lymphocytes from the patient's body in vitro, followed by the introduction of immune cells containing a retroviral vector encoding the CAR, which was then expanded extensively in vitro before re‐infusion into the patient. This time‐consuming and labor‐intensive method presents significant challenges for large‐scale implementation, as well as high costs. Recent studies have proposed the automation of CAR‐T transfection technology through micro–nano transfection techniques,^[^ ^238^ ^]^ coupled with the development of a modular universal CAR‐T cell bank,^[^ ^239^ ^]^ aiming to address the limitations of conventional CAR‐T therapy such as high costs and prolonged preparation times. Further, the cell‐surface target (such as fibroblasts) requires sufficient specificity. Fibroblasts exhibit heterogeneity both within and between tissues, and scar formation is often localized. It is advisable to minimize damage to other cells. Considering the blurred boundary between regeneration and fibrosis, the timing of CAR cell treatment is another aspect that warrants consideration. Early treatment may potentially destroy pro‐regenerative fibroblasts, leading to failure of tissue repair. Thus, future research must address two pivotal challenges: 1) leveraging single‐cell transcriptomic profiling of fibrotic niches to identify disease‐specific epitopes and enhance targeting specificity and 2) determining optimal temporal therapeutic windows to ensure intervention efficacy; while, preserving endogenous regenerative repair processes. Similar to the challenges encountered with CAR‐cell therapies in oncology, the application of CAR‐T/macrophage therapies in fibrotic diseases may also be constrained by safety concerns, particularly off‐target effects and systemic toxicities such as cytokine release syndrome (CRS). Pharmacological interventions, such as IL‐6 receptor antagonists (e.g., tocilizumab) and corticosteroids, have been employed to suppress CRS; while, low‐affinity CAR designs aim to attenuate hyperactivation.^[^ ^240^, ^241^, ^242^ ^]^ Compared to tumors, the immune cells involved in the fibrosis process are diverse, and the interaction network with other parenchymal and non‐parenchymal cells in the tissue is complex and dynamic. Unpredictable toxicities stemming from the uncontrolled behavior of CAR‐cells within fibrotic microenvironments remain a critical barrier, necessitating the development of precision control mechanisms. Moreover, the research on the role of T cells/macrophages in tissue fibrosis is not yet sufficiently deep; thus, further research is required to support the application of CAR cell therapy in fibrosis.

## 13 Future Direction

### 13.1 Trained Immunity

The innate immune system can build immunological memory, which is intricately linked to epigenetic and metabolic reprogramming. After stimulation with proinflammatory cytokines or certain ligands, such as β‐glucan and Bacille Calmette‐Guerin (BCG), these innate immune cells reshape their epigenetic modifications to acquire enhanced responding mechanisms upon restimulation, termed “trained immunity”.^[^ ^243^ ^]^ Interestingly, innate immune memory can be established in myeloid cells, innate lymphoid cells, and even stromal cells (e.g., fibroblasts and endothelial cells) and epithelial cells.^[^ ^244^, ^245^, ^246^ ^]^ It is indeed a matter of interest to consider whether the phenomenon of trained immunity, which is observed in these cells, might confer a benefit in the context of heterologous infections but could conversely pose a risk in the presence of certain endogenous stimuli, such as systemic sclerosis and chronic inflammatory stimuli. Exposure of macrophages to a high‐salt diet induces trained immunity that enhances the inflammatory response in the kidney upon subsequent exposure to LPS.^[^ ^247^ ^]^ Targeting the mTOR signaling pathway, which is critical for the development of trained immunity induced by a high‐salt diet, with the drug rapamycin, may offer a potential avenue for mitigating fibrosis associated with chronic kidney disease. A recent study shows that ischemic stroke triggers persistent innate immune memory in monocytes/macrophages and increases cardiac fibrosis and remodeling.^[^ ^248^ ^]^ This research suggests that blocking IL‐1β and monocyte trafficking after brain injury may prevent secondary organ damage. A recent study reveals that alterations in hematopoietic stem cells serve as a critical driver of recurrent heart failure and associated comorbidities, including chronic kidney disease and frailty syndrome. Integrated analysis of global chromatin accessibility landscapes and single‐cell transcriptomic profiling demonstrated compromised TGF‐β signaling within hematopoietic stem cells, which facilitated the establishment of a “stress memory” phenotype.^[^ ^249^ ^]^ This epigenetic reprogramming amplified the capacity of hematopoietic stem cells to generate pro‐inflammatory macrophages.^[^ ^249^ ^]^ This study suggests that increasing the level of TGF‐β may be a novel strategy for the treatment of recurrent heart failure and related complications. Conversely, systemic administration of the trained immunity agonist β‐glucan attenuates bleomycin‐induced pulmonary fibrosis and diminishes pulmonary collagen deposition.^[^ ^250^ ^]^ Mechanistically, this therapeutic effect is mediated through histone modification remodeling, which augments efferocytosis in alveolar macrophages and suppresses epithelial cell apoptosis via resolvin D1 release, thereby attenuating tissue injury.^[^ ^250^ ^]^ These findings highlight the therapeutic potential of prophylactically administered trained immunity agonists in fibrosis by augmenting the pro‐reparative capacity of macrophages through epigenetic reprogramming. Before advancing in the development of targeted therapies aimed at improving organ fibrosis by modulating trained immunity, it is crucial to conduct further research to elucidate the mechanisms by which innate immune memory contributes to chronic inflammatory diseases.

### 13.2 Metabolic Remodeling

The activation, differentiation, proliferation, migration, and other phenotypic and functional transformations of immune cells, as well as the development of trained immunity, are contingent upon cellular metabolic remodeling. The metabolic crosstalk between various cells within the microenvironment also constitutes an important aspect of the fibrosis process. Therefore, there has been a growing interest in the metabolic reprogramming of immune cells during the fibrosis process in recent years, making targeting the metabolic dysregulation of the immune microenvironment a new direction for the treatment of fibrotic diseases. Well‐studied macrophages are characterized by increased glycolysis and the tricarboxylic acid cycle under pro‐inflammatory conditions, whereas pro‐regenerative macrophages primarily rely on the activation of fatty acid oxidation, arginase pathway, and oxidative phosphorylation for energy provision.^[^ ^251^, ^252^ ^]^ In the murine fibrosis model, blocking the nuclear translocation of PKM2, a crucial metabolic switch in glycolysis, through inhibiting FSLT1, HSP70 family member HSPA12A, and activating Annexin A5 significantly alleviates liver fibrosis.^[^ ^253^, ^254^, ^255^ ^]^ Moreover, the inhibition of PFKFB3,^[^ ^256^ ^]^ nucleophosmin 1,^[^ ^257^ ^]^ or HIF‐1α^[^ ^258^ ^]^ suppresses glycolysis and induces metabolic, phenotypic remodeling in macrophages, which substantially improves fibrosis in liver, heart, and kidney. In arsenite‐induced hepatic fibrosis, miR‐21 accelerates the glycolysis of CD4^+^ T cells to promote CD4^+^ T‐cell polarization toward pro‐fibrotic Th2 cells through the PTEN/PI3K/AKT pathway in mice.^[^ ^259^ ^]^ The similarity in metabolic changes across different organ types points to the key pathway, glycolysis, regulating the homeostasis of the ECM and fibrosis. However, a recent study has shown that the efferocytosis capacity of regenerative macrophages needs a transient increase of glycolysis dependent on PFKFB2 activation, reminding us when targeting this metabolic pathway.^[^ ^260^ ^]^ Future work will need to integrate emerging and advanced methods, including single‐cell and spatial metabolomics, in vivo isotope tracing, and mass spectrometry imaging techniques, which will bring us multidimensional insights of cell–cell metabolic crosstalk and spatio‐temporal immune cell metabolic transition during the complex pathological process.

### 13.3 Multi‐Organ and Multi‐Omics Integration

**Figure 6:**
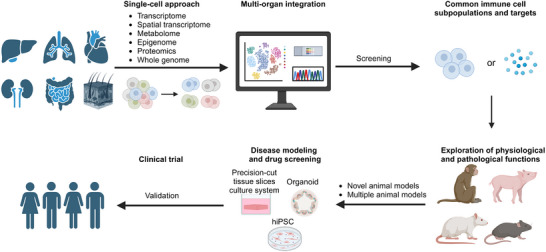
Screening therapeutic targets for fibrosis using multi‐model and multi‐omics single‐cell approaches. The pathogenesis of fibrosis is complex, involving multiple cell types within the tissue as well as cells that migrate from circulation to the tissue. Integrating single‐cell data across various temporal stages, tissues/organs, and expression levels is essential for constructing a high‐resolution spatiotemporal multi‐omics integrative atlas. This comprehensive approach facilitates the assessment of immune cell states, ontogeny, and the transitions of phenotypes and functions during human fibrotic diseases. It aims to elucidate immune cell subsets and microenvironment characteristics, as well as key biomarkers associated with the fibrotic phenotype. Further, functional validation and exploration of molecular mechanisms can be conducted using various animal models. In addition, employing precision cut tissue slices and organoid systems to develop 3D in vitro culture models can better simulate the pathological processes of fibrosis and facilitate drug screening. The novel biological insights derived from these integrative methodologies are expected to identify potential therapeutic targets. hiPSC, human induced pluripotent stem cell. Created with BioRender https://www.biorender.com/.

The transition in the composition and phenotype of immune cells within the organ from a steady state to fibrosis has begun to attract attention, with much of this analysis relying on single‐cell transcriptomics.^[^ ^261^, ^262^, ^263^ ^]^ However, comprehensive multi‐omics data integration at the single‐cell level, including transcriptome, genome, epigenome, proteome, metabolome, and spatial profiling, is still lacking. These multi‐modal single‐cell omics readouts are necessary to precisely define the immune cell state, phenotype, and function under pathological conditions, as well as the interactions between adjacent cells and the characterization of the local immune microenvironment. To excavate effective biological insights, the single‐organ multi‐omics data may not suffice to yield. Consequently, the integration of data across multiple organs could provide more information to identify novel and tractable therapeutic targets for the treatment of patients with a broad range of fibrotic diseases (**Figure** **6**).

## 14 Conclusion

Fibrosis can affect almost any organ and has become a significant global health burden. Unfortunately, to date, there is no pharmacological intervention capable of effectively reversing fibrosis. Both the innate and adaptive immune systems are integral to the fibrotic process, serving as key determinants in the ongoing chronic inflammation and its resolution. This review synthesizes recent advances in mapping the heterogeneity of immune cells within fibrotic tissues at single‐cell resolution. In addition, we explore how diverse immune cell populations respond to tissue‐specific and niche‐specific stimuli to generate effector molecules that coordinate homotypic and heterotypic cellular interactions within the microenvironment during tissue remodeling. We further summarize how alterations in the immune microenvironment influence ECM‐producing cells to acquire a pro‐fibrotic phenotype, providing novel insights into targeting immune microenvironments for fibrosis reversion. Future research should integrate spatial transcriptomics, metabolomics, epigenomics, proteomics, and genome‐wide analyses across disease spectra to map the dynamic immunological landscape from a multi‐omics, multi‐organ perspective. This approach may facilitate the identification of tractable therapeutic targets to treat patients with a broad range of fibrotic diseases. Regarding therapeutic strategies, CAR cell therapy warrants consideration though current studies remain preliminary. Further investigations are required to optimize treatment timing and mitigate potential adverse effects, including off‐target cytotoxicity and cytokine release syndrome. In conclusion, targeted manipulation of the immune microenvironment to prevent fibrotic reprogramming of injured tissues represents a vibrant and promising field in biomedical research.

## Conflict of Interest

The authors declare no conflict of interest.

## Author Contributions

X.C., C.W., and F.T. shared co‐first authorship. X.C. and J.H. conceived the manuscript. X.C. and C.W. drafted the manuscript. X.C. and F.T. drew the figures and summarized the tables. J.Z., L.M., and Y.L. revised the manuscript. All authors have read and approved the final manuscript.

## References

[1] N. C. Henderson , F. Rieder , T. A. Wynn , Nature 2020, 587, 555. 33239795 10.1038/s41586-020-2938-9 PMC8034822

[2] X. Zhao , J. Y. Y. Kwan , K. Yip , P. P. Liu , F. F. Liu , Nat. Rev. Drug Discovery 2020, 19, 57. 31548636 10.1038/s41573-019-0040-5

[3] X. Chen , H. Wang , C. Wu , X. Li , X. Huang , Y. Ren , Q. Pu , Z. Cao , X. Tang , B.‐S. Ding , Redox Biol. 2024, 70, 103038. 38266576 10.1016/j.redox.2024.103038 PMC10811458

[4] T. A. Wynn , T. R. Ramalingam , Nat. Med. 2012, 18, 1028. 22772564 10.1038/nm.2807 PMC3405917

[5] M. Zhao , L. Wang , M. Wang , S. Zhou , Y. Lu , H. Cui , A. C. Racanelli , L. Zhang , T. Ye , B. Ding , B. Zhang , J. Yang , Y. Yao , Signal Transduction Targeted Ther. 2022, 7, 206. 10.1038/s41392-022-01070-3 PMC9247101 35773269

[6] B. López , S. Ravassa , M. U. Moreno , G. S. José , J. Beaumont , A. González , J. Díez , Nat. Rev. Cardiol. 2021, 18, 479. 33568808 10.1038/s41569-020-00504-1

[7] S. J. Keam , Drugs 2024, 84, 729. 38771485 10.1007/s40265-024-02045-0

[8] S. Ryu , J. W. Shin , S. Kwon , J. Lee , Y. C. Kim , Y.‐S. Bae , Y.‐S. Bae , D. K. Kim , Y. S. Kim , S. H. Yang , H. Y. Kim , J. Clin. Invest. 2022, 132, 156876. 10.1172/JCI156876 PMC9197522 35482420

[9] J. Liu , C. Yang , T. Liu , Nat. Commun. 2020, 11, 6396. 33328477 10.1038/s41467-020-19297-5 PMC7745020

[10] S.‐S. Liu , C. Liu , X.‐X. Lv , B. Cui , J. Yan , Y.‐X. Li , K. Li , F. Hua , X.‐W. Zhang , J.‐J. Yu , J.‐M. Yu , F. Wang , S. Shang , P.‐P. Li , Z.‐G. Zhou , Y. Xiao , Z.‐W. Hu , Immunity 2021, 54, 2042. 34407391 10.1016/j.immuni.2021.06.008

[11] D. J. Baker , Z. Arany , J. A. Baur , J. A. Epstein , C. H. June , Nature 2023, 619, 707. 37495877 10.1038/s41586-023-06243-w PMC12522170

[12] K. B. R. Belchamber , L. E. Donnelly , Pharmacol. Ther. 2020, 209, 107500. 32061706 10.1016/j.pharmthera.2020.107500

[13] L. Sun , X. Wang , J. Saredy , Z. Yuan , X. Yang , H. Wang , Redox Biol. 2020, 37, 101759. 33086106 10.1016/j.redox.2020.101759 PMC7575795

[14] J. G. Rurik , H. Aghajanian , J. A. Epstein , Circ. Res. 2021, 128, 1766. 34043424 10.1161/CIRCRESAHA.121.318005 PMC8171813

[15] M. Bhattacharya , P. Ramachandran , Nat. Immunol. 2023, 24, 1423. 37474654 10.1038/s41590-023-01551-9

[16] T. Lazarov , S. Juarez‐Carreno , N. Cox , F. Geissmann , Nature 2023, 618, 698. 37344646 10.1038/s41586-023-06002-x PMC10649266

[17] P. Rodriguez‐Morales , R. A. Franklin , Trends Immunol. 2023, 44, 986. 37940394 10.1016/j.it.2023.10.004 PMC10841626

[18] P. J. Murray , Annu. Rev. Physiol. 2017, 79, 541. 27813830 10.1146/annurev-physiol-022516-034339

[19] C. He , A. J. Ryan , S. Murthy , A. B. Carter , J. Biol. Chem. 2013, 288, 20745. 23720777 10.1074/jbc.M112.410720 PMC3711337

[20] S. Su , Q. Zhao , C. He , D. Huang , J. Liu , F. Chen , J. Chen , J.‐Y. Liao , X. Cui , Y. Zeng , H. Yao , F. Su , Q. Liu , S. Jiang , E. Song , Nat. Commun. 2015, 6, 8523. 26436920 10.1038/ncomms9523 PMC4600756

[21] X. Bao , X. Liu , N. Liu , S. Zhuang , Q. Yang , H. Ren , D. Zhao , J. Bai , X. Zhou , L. Tang , Respir. Res. 2021, 22, 194. 34217280 10.1186/s12931-021-01785-x PMC8255011

[22] J. Zhang , Y. Liu , H. Chen , Q. Yuan , J. Wang , M. Niu , L. Hou , J. Gu , J. Zhang , Cell Death Dis. 2022, 13, 411. 35484116 10.1038/s41419-022-04802-z PMC9051099

[23] D. Aran , A. P. Looney , L. Liu , E. Wu , V. Fong , A. Hsu , S. Chak , R. P. Naikawadi , P. J. Wolters , A. R. Abate , A. J. Butte , M. Bhattacharya , Nat. Immunol. 2019, 20, 163. 30643263 10.1038/s41590-018-0276-y PMC6340744

[24] T. Fabre , A. M. S. Barron , S. M. Christensen , S. Asano , K. Bound , M. P. Lech , M. H. Wadsworth , X. Chen , C. Wang , J. Wang , J. McMahon , F. Schlerman , A. White , K. M. Kravarik , A. J. Fisher , L. A. Borthwick , K. M. Hart , N. C. Henderson , T. A. Wynn , K. Dower , Sci. Immunol. 2023, 8, add8945. 10.1126/sciimmunol.add8945 37027478

[25] J. Wang , M. Jiang , A. Xiong , L. Zhang , L. Luo , Y. Liu , S. Liu , Q. Ran , D. Wu , Y. Xiong , X. He , E. L.‐H. Leung , G. Li , Pharmacol. Res. 2022, 182, 106286. 35662628 10.1016/j.phrs.2022.106286

[26] C. Morse , T. Tabib , J. Sembrat , K. L. Buschur , H. T. Bittar , E. Valenzi , Y. Jiang , D. J. Kass , K. Gibson , W. Chen , A. Mora , P. V. Benos , M. Rojas , R. Lafyatis , Eur. Respir. J. 2019, 54, 1802441. 31221805 10.1183/13993003.02441-2018 PMC8025672

[27] K. Hoeft , G. J. L. Schaefer , H. Kim , D. Schumacher , T. Bleckwehl , Q. Long , B. M. Klinkhammer , F. Peisker , L. Koch , J. Nagai , M. Halder , S. Ziegler , E. Liehn , C. Kuppe , J. Kranz , S. Menzel , I. Costa , A. Wahida , P. Boor , R. K. Schneider , S. Hayat , R. Kramann , Cell Rep. 2023, 42, 112131. 36807143 10.1016/j.celrep.2023.112131 PMC9992450

[28] A. V. Misharin , L. Morales‐Nebreda , P. A. Reyfman , C. M. Cuda , J. M. Walter , A. C. McQuattie‐Pimentel , C.‐I. Chen , K. R. Anekalla , N. Joshi , K. J. N. Williams , H. Abdala‐Valencia , T. J. Yacoub , M. Chi , S. Chiu , F. J. Gonzalez‐Gonzalez , K. Gates , A. P. Lam , T. T. Nicholson , P. J. Homan , S. Soberanes , S. Dominguez , V. K. Morgan , R. Saber , A. Shaffer , M. Hinchcliff , S. A. Marshall , A. Bharat , S. Berdnikovs , S. M. Bhorade , E. T. Bartom , et al., J. Exp. Med. 2017, 214, 2387. 28694385 10.1084/jem.20162152 PMC5551573

[29] N. Joshi , S. Watanabe , R. Verma , R. P. Jablonski , C.‐I. Chen , P. Cheresh , N. S. Markov , P. A. Reyfman , A. C. McQuattie‐Pimentel , L. Sichizya , Z. Lu , R. Piseaux‐Aillon , D. Kirchenbuechler , A. S. Flozak , C. J. Gottardi , C. M. Cuda , H. Perlman , M. Jain , D. W. Kamp , G. R. S. Budinger , A. V. Misharin , Eur. Respir. J. 2020, 55, 1900646. 31601718 10.1183/13993003.00646-2019 PMC6962769

[30] L. Meziani , M. Mondini , B. Petit , A. Boissonnas , V. Thomas de Montpreville , O. Mercier , M.‐C. Vozenin , E. Deutsch , Eur. Respir. J. 2018, 51, 1702120. 29496785 10.1183/13993003.02120-2017

[31] J. Saris , A. Y. F. Li Yim , S. Bootsma , K. J. Lenos , R. Franco Fernandez , H. N. Khan , J. Verhoeff , D. Poel , N. M. Mrzlikar , L. Xiong , M. P. Schijven , N. C. T. van Grieken , O. Kranenburg , M. E. Wildenberg , A. Logiantara , C. Jongerius , J. J. Garcia Vallejo , S. S. Gisbertz , S. Derks , J. B. Tuynman , G. R. A. M. D'Haens , L. Vermeulen , J. Grootjans , Nat. Commun. 2025, 16, 3669. 40246872 10.1038/s41467-025-58999-6 PMC12006467

[32] T. Hendrikx , F. Porsch , M. G. Kiss , D. Rajcic , N. Papac‐Milicevic , C. Hoebinger , L. Goederle , A. Hladik , L. E. Shaw , H. Horstmann , S. Knapp , S. Derdak , M. Bilban , L. Heintz , M. Krawczyk , R. Paternostro , M. Trauner , M. Farlik , D. Wolf , C. J. Binder , J. Hepatol. 2022, 77, 1373. 35750138 10.1016/j.jhep.2022.06.004

[33] H. Y. Li , S. W. Fu , J. C. Wu , Z. H. Li , M. Y. Xu , Inflammation Res. 2023, 72, 669. 10.1007/s00011-023-01696-1 36745210

[34] Y.‐H. Li , S. Shen , T. Shao , M.‐T. Jin , D.‐D. Fan , A.‐F. Lin , L.‐X. Xiang , J.‐Z. Shao , Cell Death Discovery 2021, 7, 239. 34518510 10.1038/s41420-021-00584-z PMC8437974

[35] P. Ramachandran , A. Pellicoro , M. A. Vernon , L. Boulter , R. L. Aucott , A. Ali , S. N. Hartland , V. K. Snowdon , A. Cappon , T. T. Gordon‐Walker , M. J. Williams , D. R. Dunbar , J. R. Manning , N. van Rooijen , J. A. Fallowfield , S. J. Forbes , J. P. Iredale , Proc. Natl. Acad. Sci. U. S. A. 2012, 109, E3186. 23100531 10.1073/pnas.1119964109 PMC3503234

[36] C. Büttner , A. Skupin , T. Reimann , E. P. Rieber , G. Unteregger , P. Geyer , K.‐H. Frank , Am. J. Respir. Cell Mol. Biol. 1997, 17, 315. 9308918 10.1165/ajrcmb.17.3.2279

[37] L. Su , Y. Dong , Y. Wang , Y. Wang , B. Guan , Y. Lu , J. Wu , X. Wang , D. Li , A. Meng , F. Fan , Cell Death Dis. 2021, 12, 527. 34023858 10.1038/s41419-021-03811-8 PMC8141056

[38] S. Li , B. Zhou , M. Xue , J. Zhu , G. Tong , J. Fan , K. Zhu , Z. Hu , R. Chen , Y. Dong , Y. Chen , K. Y. Lee , X. Li , L. Jin , W. Cong , Hepatology 2023, 77, 816. 35753047 10.1002/hep.32640

[39] J. Chen , Q. Liu , J. He , Y. Li , Front. Immunol. 2022, 13, 958790. 36045667 10.3389/fimmu.2022.958790 PMC9420855

[40] P. Pakshir , M. Alizadehgiashi , B. Wong , N. M. Coelho , X. Chen , Z. Gong , V. B. Shenoy , C. A. McCulloch , B. Hinz , Nat. Commun. 2019, 10, 1850. 31015429 10.1038/s41467-019-09709-6 PMC6478854

[41] A. J. Thorley , P. A. Ford , M. A. Giembycz , P. Goldstraw , A. Young , T. D. Tetley , J. Immunol. 2007, 178, 463. 17182585 10.4049/jimmunol.178.1.463

[42] A. Papazoglou , M. Huang , M. Bulik , A. Lafyatis , T. Tabib , C. Morse , J. Sembrat , M. Rojas , E. Valenzi , R. Lafyatis , Arthritis Rheumatol. 2022, 74, 2003. 35849803 10.1002/art.42286 PMC9771864

[43] L. Liu , X. Jin , C.‐F. Hu , Y.‐P. Zhang , Z.'e Zhou , R. Li , C.‐X. Shen , Basic Res. Cardiol. 2018, 113, 12. 29349588 10.1007/s00395-018-0669-y

[44] M. J. Perugorria , M. U. Latasa , A. Nicou , H. Cartagena‐Lirola , J. Castillo , S. Goñi , U. Vespasiani‐Gentilucci , M. G. Zagami , S. Lotersztajn , J. Prieto , C. Berasain , M. A. Avila , Hepatology 2008, 48, 1251. 18634036 10.1002/hep.22437

[45] L. Ding , T. Liu , Z. Wu , B. Hu , T. Nakashima , M. Ullenbruch , F. Gonzalez De Los Santos , S. H. Phan , J. Immunol. 2016, 197, 303. 27206766 10.4049/jimmunol.1502479 PMC4912903

[46] C. M. Minutti , R. V. Modak , F. Macdonald , F. Li , D. J. Smyth , D. A. Dorward , N. Blair , C. Husovsky , A. Muir , E. Giampazolias , R. Dobie , R. M. Maizels , T. J. Kendall , D. W. Griggs , M. Kopf , N. C. Henderson , D. M. Zaiss , Immunity 2019, 50, 645. 30770250 10.1016/j.immuni.2019.01.008 PMC6436929

[47] J. H. Ko , H. J. Kim , H. J. Jeong , H. J. Lee , J. Y. Oh , Cell Rep. 2020, 30, 3806. 32187551 10.1016/j.celrep.2020.02.062

[48] N. Nacu , I. G. Luzina , K. Highsmith , V. Lockatell , K. Pochetuhen , Z. A. Cooper , M. P. Gillmeister , N. W. Todd , S. P. Atamas , J. Immunol. 2008, 180, 5036. 18354229 10.4049/jimmunol.180.7.5036 PMC2847349

[49] H. Fu , Y. H. Gu , J. Tan , Y. N. Yang , G. H. Wang , Cell. Mol. Life Sci. 2022, 79, 253. 35449370 10.1007/s00018-022-04247-9 PMC11072867

[50] L. Alonso‐Herranz , A. Sahun‐Espanol , A. Paredes , Elife 2020, 9, 57920. 10.7554/eLife.57920 PMC7609061 33063665

[51] X. Ge , P. Tang , Y. Rong , D. Jiang , X. Lu , C. Ji , J. Wang , C. Huang , A. Duan , Y. Liu , X. Chen , X. Chen , Z. Xu , F. Wang , Z. Wang , X. Li , W. Zhao , J. Fan , W. Liu , G. Yin , W. Cai , Redox Biol. 2021, 41, 101932. 33714739 10.1016/j.redox.2021.101932 PMC7967037

[52] B. Charreau , Transplantation 2021, 105, 156. 10.1097/TP.0000000000003741 33724240

[53] F. Iavarone , O. Guardiola , A. Scagliola , EMBO Rep. 2020, 21, 49075. 10.15252/embr.201949075 PMC7132341 32107853

[54] P. Zordan , E. Rigamonti , K. Freudenberg , V. Conti , E. Azzoni , P. Rovere‐Querini , S. Brunelli , Cell Death Dis. 2014, 5, 1031. 10.1038/cddis.2013.558 PMC4040684 24481445

[55] P. M.‐K. Tang , Y.‐Y. Zhang , J. Xiao , P. C.‐T. Tang , J. Y.‐F. Chung , J. Li , V. W. Xue , X.‐R. Huang , C. C.‐N. Chong , C.‐F. Ng , T.‐L. Lee , K.‐F. To , D. J. Nikolic‐Paterson , H.‐Y. Lan , Proc. Natl. Acad. Sci. U. S. A. 2020, 117, 20741. 32788346 10.1073/pnas.1917663117 PMC7456094

[56] N. Haider , L. Boscá , H. R. Zandbergen , J. C. Kovacic , N. Narula , S. González‐Ramos , M. Fernandez‐Velasco , S. Agrawal , M. Paz‐García , S. Gupta , K. DeLeon‐Pennell , V. Fuster , B. Ibañez , J. Narula , J. Am. Coll. Cardiol. 2019, 74, 3124. 31856969 10.1016/j.jacc.2019.10.036 PMC7425814

[57] X. Huang , C. He , X. Hua , A. Kan , Y. Mao , S. Sun , F. Duan , J. Wang , P. Huang , S. Li , Clin. Transl. Med. 2020, 10, 41. 10.1002/ctm2.41 PMC7403727 32508052

[58] Y.‐Y. Wang , H. Jiang , J. Pan , X.‐R. Huang , Y.‐C. Wang , H.‐F. Huang , K.‐F. To , D. J. Nikolic‐Paterson , H.‐Y. Lan , J.‐H. Chen , J. Am. Soc. Nephrol. 2017, 28, 2053. 28209809 10.1681/ASN.2016050573 PMC5491278

[59] J. Chen , Y. Tang , Y. Zhong , B. Wei , X.‐R. Huang , P. M.‐K. Tang , A. Xu , H.‐Y. Lan , Mol. Ther. 2022, 30, 3017. 35791881 10.1016/j.ymthe.2022.06.019 PMC9481993

[60] P. M.‐K. Tang , S. Zhou , C.‐J. Li , J. Liao , J. Xiao , Q.‐M. Wang , G.‐Y. Lian , J. Li , X.‐R. Huang , K.‐F. To , C.‐F. NG , C. C.‐N. Chong , R. C.‐W. Ma , T.‐L. Lee , H.‐Y. Lan , Kidney Int. 2018, 93, 173. 29042082 10.1016/j.kint.2017.07.026

[61] Y. Wang , Y. Li , Z. Chen , Y. Yuan , Q. Su , K. Ye , C. Chen , G. Li , Y. Song , H. Chen , Y. Xu , Cell Death Dis. 2022, 13, 693. 35941120 10.1038/s41419-022-05138-4 PMC9360039

[62] L. Luo , S. Wang , Y. Hu , L. Wang , X. Jiang , J. Zhang , X. Liu , X. Guo , Z. Luo , C. Zhu , M. Xie , Y. Li , J. You , F. Yang , ACS Nano 2023, 17, 22508. 37948096 10.1021/acsnano.3c05998

[63] V. J. Craig , F. Polverino , M. E. Laucho‐Contreras , Y. Shi , Y. Liu , J. C. Osorio , Y. Tesfaigzi , V. Pinto‐Plata , B. R. Gochuico , I. O. Rosas , C. A. Owen , PLoS One 2014, 9, 97485. 10.1371/journal.pone.0097485 PMC4020836 24828408

[64] V. J. Craig , L. Zhang , J. S. Hagood , C. A. Owen , Am. J. Respir. Cell Mol. Biol. 2015, 53, 585. 26121236 10.1165/rcmb.2015-0020TR PMC4742954

[65] X. Zhou , C. Zhang , S. Yang , L. Yang , W. Luo , W. Zhang , X. Zhang , J. Chao , J. Hazard. Mater. 2024, 461, 132733. 37816293 10.1016/j.jhazmat.2023.132733

[66] M. Y. Yao , W. H. Zhang , W. T. Ma , Q. H. Liu , L. H. Xing , G. F. Zhao , Exp. Mol. Med. 2019, 51, 1. 10.1038/s12276-019-0255-x PMC6547742 31164635

[67] D. Wang , C. Hao , L. Zhang , J. Zhang , S. Liu , Y. Li , Y. Qu , Y. Zhao , R. Huang , J. Wei , W. Yao , Ecotoxicol. Environ. Saf. 2020, 192, 110253. 32059163 10.1016/j.ecoenv.2020.110253

[68] C. Wang , C. Zhang , L. Liu , A. Xi , B. Chen , Y. Li , J. Du , Mol. Ther. 2017, 25, 192. 28129114 10.1016/j.ymthe.2016.09.001 PMC5363311

[69] B. Shu , R. Z. Zhang , Y. X. Zhou , C. He , X. Yang , Cell Death Discovery 2022, 8, 266. 35585044 10.1038/s41420-022-01036-y PMC9117676

[70] M. G. Rohani , R. S. McMahan , M. V. Razumova , A. L. Hertz , M. Cieslewicz , S. H. Pun , M. Regnier , Y. Wang , T. P. Birkland , W. C. Parks , J. Invest. Dermatol. 2015, 135, 2377. 25927164 10.1038/jid.2015.167 PMC4567949

[71] J. Ren , J. Zhang , N. P. Rudemiller , R. Griffiths , Y. Wen , X. Lu , J. R. Privratsky , M. D. Gunn , S. D. Crowley , J. Am. Soc. Nephrol. 2019, 30, 1674. 31315922 10.1681/ASN.2018121253 PMC6727252

[72] J. Guiot , M. Cambier , A. Boeckx , M. Henket , O. Nivelles , F. Gester , E. Louis , M. Malaise , F. Dequiedt , R. Louis , I. Struman , M.‐S. Njock , Thorax 2020, 75, 870. 32759383 10.1136/thoraxjnl-2019-214077 PMC7509395

[73] Z. Wan , X. Yang , X. Liu , iScience 2022, 25, 104597. 35789846 10.1016/j.isci.2022.104597 PMC9249826

[74] X. Hou , S. Yin , R. Ren , S. Liu , L. Yong , Y. Liu , Y. Li , M.‐H. Zheng , G. Kunos , B. Gao , H. Wang , Hepatology 2021, 74, 116. 33236445 10.1002/hep.31658 PMC8141545

[75] S. Bhatnagar , K. Shinagawa , F. J. Castellino , J. S. Schorey , Blood 2007, 110, 3234. 17666571 10.1182/blood-2007-03-079152 PMC2200902

[76] J. Villar , E. Segura , Trends Immunol. 2020, 41, 1062. 33250080 10.1016/j.it.2020.10.002

[77] C. Kurts , F. Ginhoux , U. Panzer , Nat. Rev. Nephrol. 2020, 16, 391. 32372062 10.1038/s41581-020-0272-y

[78] S. Sutti , I. Locatelli , S. Bruzzì , A. Jindal , M. Vacchiano , C. Bozzola , E. Albano , Clin. Sci. 2015, 129, 797. 10.1042/CS20150053 26253086

[79] R. L. Ross , C. Corinaldesi , G. Migneco , I. M. Carr , A. Antanaviciute , C. W. Wasson , A. Carriero , J. H. W. Distler , S. Holmes , Y. M. El‐Sherbiny , C. S. McKimmie , F. Del Galdo , Ann. Rheum. Dis. 2021, 80, 920. 33542104 10.1136/annrheumdis-2020-218439 PMC8237203

[80] M. D. Ah Kioon , C. Tripodo , D. Fernandez , K. A. Kirou , R. F. Spiera , M. K. Crow , J. K. Gordon , F. J. Barrat , Sci. Transl. Med. 2018, 10, aam8458. 10.1126/scitranslmed.aam8458 PMC9865429 29321259

[81] J. A. Choreño‐Parra , D. Cervantes‐Rosete , L. A. Jiménez‐Álvarez , G. Ramírez‐Martínez , J. E. Márquez‐García , A. Cruz‐Lagunas , A. Y. Magaña‐Sánchez , G. Lima , H. López‐Maldonado , E. Gaytán‐Guzmán , A. Caballero , R. Fernández‐Plata , J. Furuzawa‐Carballeda , C. Mendoza‐Milla , M. D. C. Navarro‐González , L. Llorente , J. Ziga , T. S. Rodríguez‐Reyna , Rheumatology 2023, 62, 1687. 36063053 10.1093/rheumatology/keac489 PMC10070068

[82] D. Fang , B. Chen , A. Lescoat , D. Khanna , R. Mu , Nat. Rev. Rheumatol. 2022, 18, 683. 36352098 10.1038/s41584-022-00864-7

[83] V. Lukacs‐Kornek , D. Schuppan , J. Hepatol. 2013, 59, 1124. 23727306 10.1016/j.jhep.2013.05.033

[84] J. Jiao , D. Sastre , M. I. Fiel , U. E. Lee , Z. Ghiassi‐Nejad , F. Ginhoux , E. Vivier , S. L. Friedman , M. Merad , C. Aloman , Hepatology 2012, 55, 244. 21898476 10.1002/hep.24621 PMC3245345

[85] Y. Xu , F. Liu , D. He , L. Han , X. Zheng , M. Hu , P. Chen , Immunobiology 2023, 228, 152315. 36608595 10.1016/j.imbio.2022.152315

[86] M. B. Khawar , F. Azam , N. Sheikh , K. Abdul Mujeeb , J. Immunol. Res. 2016, 8, 2148129. 10.1155/2016/2148129 PMC5168458 28050571

[87] K. Wang , J. Wang , M. Song , H. Wang , N. Xia , Y. Zhang , Int. J. Biol. Macromol. 2020, 162, 273. 32569681 10.1016/j.ijbiomac.2020.06.166

[88] I. D. Odell , H. Steach , S. B. Gauld , L. Reinke‐Breen , J. Karman , T. L. Carr , J. B. Wetter , L. Phillips , M. Hinchcliff , R. A. Flavell , Sci. Immunol. 2022, 7, abq6691. 10.1126/sciimmunol.abq6691 PMC9840167 36490328

[89] K. T. K. Giuliani , A. Grivei , P. Nag , X. Wang , M. Rist , K. Kildey , B. Law , M. S. Ng , R. Wilkinson , J. Ungerer , J. M. Forbes , H. Healy , A. J. Kassianos , Cell Death Dis. 2022, 13, 739. 36030251 10.1038/s41419-022-05191-z PMC9420140

[90] M. Xiang , T. Liu , C. Tian , K. Ma , J. Gou , R. Huang , S. Li , Q. Li , C. Xu , L. Li , C.‐H. Lee , Y. Zhang , Pharmacol. Res. 2022, 177, 106092. 35066108 10.1016/j.phrs.2022.106092 PMC8776354

[91] J. J. Chia , T. Zhu , S. Chyou , D. C. Dasoveanu , C. Carballo , S. Tian , C. M. Magro , S. Rodeo , R. F. Spiera , N. H. Ruddle , T. E. McGraw , J. L. Browning , R. Lafyatis , J. K. Gordon , T. T. Lu , J. Clin. Invest. 2016, 126, 4331. 27721238 10.1172/JCI85740 PMC5096920

[92] S. M. Blois , F. Piccioni , N. Freitag , I. Tirado‐González , P. Moschansky , R. Lloyd , K. Hensel‐Wiegel , M. Rose , M. G. Garcia , L. D. Alaniz , G. Mazzolini , Angiogenesis 2014, 17, 119. 24068342 10.1007/s10456-013-9382-5

[93] P. X. Liew , P. Kubes , Physiol. Rev. 2019, 99, 1223. 30758246 10.1152/physrev.00012.2018

[94] M. Phillipson , P. Kubes , Trends Immunol. 2019, 40, 635. 31160208 10.1016/j.it.2019.05.001

[95] H. Guo , S. Chen , M. Xie , C. Zhou , M. Zheng , Cell Prolif. 2021, 54, 13040. 10.1111/cpr.13040 PMC8168408 33942422

[96] L. G. Ng , R. Ostuni , A. Hidalgo , Nat. Rev. Immunol. 2019, 19, 255. 30816340 10.1038/s41577-019-0141-8

[97] D. Hartl , S. Krauss‐Etschmann , B. Koller , P. L. Hordijk , T. W. Kuijpers , F. Hoffmann , A. Hector , E. Eber , V. Marcos , I. Bittmann , O. Eickelberg , M. Griese , D. Roos , J. Immunol. 2008, 181, 8053. 19017998 10.4049/jimmunol.181.11.8053

[98] K. T. Mincham , N. Bruno , A. Singanayagam , R. J. Snelgrove , Immunology 2021, 164, 701. 34547115 10.1111/imm.13419 PMC8561104

[99] H. Wang , M. Gao , J. Li , J. Sun , R. Wu , D. Han , J. Tan , J. Wang , B. Wang , L. Zhang , Y. Dong , Acta Physiol. 2019, 227, 13317. 10.1111/apha.13317 31132220

[100] E. Vafadarnejad , G. Rizzo , L. Krampert , P. Arampatzi , A.‐P. Arias‐Loza , Y. Nazzal , A. Rizakou , T. Knochenhauer , S. R. Bandi , V. A. Nugroho , D. J. J. Schulz , M. Roesch , P. Alayrac , J. Vilar , J.‐S. Silvestre , A. Zernecke , A.‐E. Saliba , C. Cochain , Circ. Res. 2020, 127, 232. 10.1161/CIRCRESAHA.120.317200 32811295

[101] E. Martini , P. Kunderfranco , C. Peano , P. Carullo , M. Cremonesi , T. Schorn , R. Carriero , A. Termanini , F. S. Colombo , E. Jachetti , C. Panico , G. Faggian , A. Fumero , L. Torracca , M. Molgora , J. Cibella , C. Pagiatakis , J. Brummelman , G. Alvisi , E. M. C. Mazza , M. P. Colombo , E. Lugli , G. Condorelli , M. Kallikourdis , Circulation 2019, 140, 2089. 31661975 10.1161/CIRCULATIONAHA.119.041694

[102] A. Nishimoto‐Kakiuchi , I. Sato , K. Nakano , H. Ohmori , Y. Kayukawa , H. Tanimura , S. Yamamoto , Y. Sakamoto , G. Nakamura , A. Maeda , K. Asanuma , A. Kato , T. Sankai , R. Konno , H. Yamada‐Okabe , Sci. Transl. Med. 2023, 15, abq5858. 10.1126/scitranslmed.abq5858 36812343

[103] T. Fabre , M. F. Molina , G. Soucy , J.‐P. Goulet , B. Willems , J.‐P. Villeneuve , M. Bilodeau , N. H. Shoukry , Sci. Immunol. 2018, 3, aar7754. 10.1126/sciimmunol.aar7754 30366940

[104] F. Yang , H. Li , Y. Li , Y. Hao , C. Wang , P. Jia , X. Chen , S. Ma , Z. Xiao , Int. Immunopharmacol. 2021, 99, 108051. 34426110 10.1016/j.intimp.2021.108051

[105] A. Fischer , J. Wannemacher , S. Christ , T. Koopmans , S. Kadri , J. Zhao , M. Gouda , H. Ye , M. Mück‐Häusl , P. W. Krenn , H.‐G. Machens , R. Fässler , P.‐A. Neumann , S. M. Hauck , Y. Rinkevich , Nat. Immunol. 2022, 23, 518. 35354953 10.1038/s41590-022-01166-6 PMC8986538

[106] B. Kaufmann , A. Leszczynska , A. Reca , L. M. Booshehri , J. Onyuru , Z. Tan , A. Wree , H. Friess , D. Hartmann , B. Papouchado , L. Broderick , H. M. Hoffman , B. A. Croker , Y. P. Zhu , A. E. Feldstein , EMBO Rep. 2022, 23, 54446. 10.15252/embr.202154446 PMC9638850 36194627

[107] Z. Ren , K. Yang , M. Zhao , W. Liu , X. Zhang , J. Chi , Z. Shi , X. Zhang , Y. Fu , Y. Liu , X. Yin , Can. J. Cardiol. 2020, 36, 893. 32224080 10.1016/j.cjca.2019.09.026

[108] W. Zeng , Y. Song , R. Wang , R. He , T. Wang , J. Pharm. Anal. 2023, 13, 355. 37181292 10.1016/j.jpha.2022.12.003 PMC10173178

[109] L. Zhou , R. Gao , H. Hong , X. Li , J. Yang , W. Shen , Z. Wang , J. Yang , J. Cell. Mol. Med. 2020, 24, 11998. 32935466 10.1111/jcmm.15827 PMC7578861

[110] Y. Ogura , K. Tajiri , N. Murakoshi , D. Xu , S. Yonebayashi , S. Li , Y. Okabe , D. Feng , Y. Shimoda , Z. Song , H. Mori , Z. Yuan , K. Aonuma , M. Ieda , Int. J. Mol. Sci. 2021, 22, 722. 33450865 10.3390/ijms22020722 PMC7828348

[111] M. Elias , S. Zhao , H. T. Le , J. Wang , M. F. Neurath , C. Neufert , C. Fiocchi , F. Rieder , J. Clin. Invest. 2021, 131, 144336. 33463541 10.1172/JCI144336 PMC7810483

[112] K. M. Lodge , A. Vassallo , B. Liu , M. Long , Z. Tong , P. R. Newby , D. Agha‐Jaffar , K. Paschalaki , C. E. Green , K. B. R. Belchamber , V. C. Ridger , R. A. Stockley , E. Sapey , C. Summers , A. S. Cowburn , E. R. Chilvers , W. Li , A. M. Condliffe , Am J. Respir. Crit. Care Med. 2022, 205, 903. 35044899 10.1164/rccm.202006-2467OC PMC9838628

[113] S. Sharma , T. M. Hofbauer , A. S. Ondracek , Blood 2021, 137, 1104. 33512471 10.1182/blood.2020005861

[114] T. M. Hofbauer , A. Mangold , T. Scherz , V. Seidl , A. Panzenböck , A. S. Ondracek , J. Müller , M. Schneider , T. Binder , L. Hell , I. M. Lang , Basic Res. Cardiol. 2019, 114, 33. 31312919 10.1007/s00395-019-0740-3 PMC6647191

[115] W. Wang , Z. Liu , Y. Zhang , L. Wang , D. Meng , X. Li , J. Zhang , Y. Wu , X. Zhou , G. Liu , Environ. Pollut. 2022, 309, 119743. 35835272 10.1016/j.envpol.2022.119743

[116] L. Pandolfi , S. Bozzini , V. Frangipane , E. Percivalle , A. De Luigi , M. B. Violatto , G. Lopez , E. Gabanti , L. Carsana , M. D'Amato , M. Morosini , M. De Amici , M. Nebuloni , T. Fossali , R. Colombo , L. Saracino , V. Codullo , M. Gnecchi , P. Bigini , F. Baldanti , D. Lilleri , F. Meloni , Front. Immunol. 2021, 12, 663303. 34194429 10.3389/fimmu.2021.663303 PMC8236949

[117] Z. Zhang , S. Ding , Z. Wang , X. Zhu , Z. Zhou , W. Zhang , X. Yang , J. Ge , Acta Pharm. Sin. B 2022, 12, 1840. 35847488 10.1016/j.apsb.2021.10.016 PMC9279636

[118] M. Negreros , L. F. Flores‐Suarez , Autoimmun Rev. 2021, 20, 102781. 33609801 10.1016/j.autrev.2021.102781

[119] S. Zhang , X. Jia , Q. Zhang , L. Zhang , J. Yang , C. Hu , J. Shi , X. Jiang , J. Lu , H. Shen , J. Cell. Mol. Med. 2020, 24, 1658. 31821687 10.1111/jcmm.14858 PMC6991674

[120] A. Mousset , E. Lecorgne , I. Bourget , P. Lopez , K. Jenovai , J. Cherfils‐Vicini , C. Dominici , G. Rios , C. Girard‐Riboulleau , B. Liu , D. L. Spector , S. Ehmsen , S. Renault , C. Hego , F. Mechta‐Grigoriou , F.‐C. Bidard , M. G. Terp , M. Egeblad , C. Gaggioli , J. Albrengues , Cancer Cell 2023, 41, 757. 37037615 10.1016/j.ccell.2023.03.008 PMC10228050

[121] E. Frangou , A. Chrysanthopoulou , A. Mitsios , K. Kambas , S. Arelaki , I. Angelidou , A. Arampatzioglou , H. Gakiopoulou , G. K. Bertsias , P. Verginis , K. Ritis , D. T. Boumpas , Ann. Rheum. Dis. 2019, 78, 238. 30563869 10.1136/annrheumdis-2018-213181 PMC6352428

[122] R. Yadav , A. Momin , C. Godugu , Int. Immunopharmacol. 2023, 124, 110846. 37634446 10.1016/j.intimp.2023.110846

[123] C. J. Calvente , M. Tameda , C. D. Johnson , H. del Pilar , Y. C. Lin , N. Adronikou , X. De Mollerat Du Jeu , C. Llorente , J. Boyer , A. E. Feldstein , J. Clin. Invest. 2019, 129, 4091. 31295147 10.1172/JCI122258 PMC6763256

[124] E. Saijou , Y. Enomoto , M. Matsuda , C. Yuet‐Yin Kok , S. Akira , M. Tanaka , A. Miyajima , Hepatol. Commun. 2018, 2, 703. 29881822 10.1002/hep4.1178 PMC5983199

[125] J. Blázquez‐Prieto , I. López‐Alonso , L. Amado‐Rodríguez , C. Huidobro , A. González‐López , W. M. Kuebler , G. M. Albaiceta , Thorax 2018, 73, 321. 28947666 10.1136/thoraxjnl-2017-210105

[126] M. Horckmans , L. Ring , J. Duchene , D. Santovito , M. J. Schloss , M. Drechsler , C. Weber , O. Soehnlein , S. Steffens , Eur. Heart J. 2017, 38, 187. 28158426 10.1093/eurheartj/ehw002

[127] W. Yang , Y. Tao , Y. Wu , X. Zhao , W. Ye , D. Zhao , L. Fu , C. Tian , J. Yang , F. He , L. Tang , Nat. Commun. 2019, 10, 1076. 30842418 10.1038/s41467-019-09046-8 PMC6403250

[128] J. Tai?eb , C. Delarche , V. Paradis , P. Mathurin , A. Grenier , B. Crestani , M. Dehoux , D. Thabut , M.‐A. Gougerot‐Pocidalo , T. Poynard , S. Chollet‐Martin , J. Hepatol. 2002, 36, 342. 11867177 10.1016/s0168-8278(01)00276-8

[129] M. Siwicki , P. Kubes , J. Allergy Clin. Immunol. 2023, 151, 634. 36642653 10.1016/j.jaci.2022.12.004

[130] S. Wernersson , G. Pejler , Nat. Rev. Immunol. 2014, 14, 478. 24903914 10.1038/nri3690

[131] D. Voehringer , Nat. Rev. Immunol. 2013, 13, 362. 23558889 10.1038/nri3427

[132] K. F. Rabe , S. Rennard , F. J. Martinez , B. R. Celli , D. Singh , A. Papi , M. Bafadhel , J. Heble , A. Radwan , X. Soler , J. A. Jacob Nara , Y. Deniz , P. J. Rowe , Am J. Respir. Crit. Care Med. 2023, 208, 395. 37348121 10.1164/rccm.202303-0455CI

[133] R. L. Gieseck 3rd , M. S. Wilson , T. A. Wynn , Nat. Rev. Immunol. 2018, 18, 62. 28853443 10.1038/nri.2017.90

[134] F. Sicklinger , I. S. Meyer , X. Li , D. Radtke , S. Dicks , M. P. Kornadt , C. Mertens , J. K. Meier , K. J. Lavine , Y. Zhang , T. C. Kuhn , T. Terzer , J. Patel , M. Boerries , G. Schramm , N. Frey , H. A. Katus , D. Voehringer , F. Leuschner , J. Clin. Invest. 2021, 131, 136778. 10.1172/JCI136778 PMC8245180 34196299

[135] G. Schiechl , F. J. Hermann , M. Rodriguez Gomez , S. Kutzi , K. Schmidbauer , Y. Talke , S. Neumayer , N. Goebel , K. Renner , H. Brühl , H. Karasuyama , K. Obata‐Ninomiya , K. Utpatel , M. Evert , S. W. Hirt , E. K. Geissler , S. Fichtner‐Feigl , M. Mack , Am. J. Transplant. 2016, 16, 2574. 26932231 10.1111/ajt.13764

[136] P. Conti , A. Caraffa , F. Mastrangelo , L. Tettamanti , G. Ronconi , I. Frydas , S. K. Kritas , T. C. Theoharides , Cell Prolif. 2018, 51, 12475. 10.1111/cpr.12475 PMC6528914 30062695

[137] M. Hara , A. Matsumori , K. Ono , H. Kido , M.‐W. Hwang , T. Miyamoto , A. Iwasaki , M. Okada , K. Nakatani , S. Sasayama , Circulation 1999, 100, 1443. 10500047 10.1161/01.cir.100.13.1443

[138] A. C. Reid , R. B. Silver , R. Levi , Immunol. Rev. 2007, 217, 123. 17498056 10.1111/j.1600-065X.2007.00514.x

[139] J. Janicki , G. Brower , J. Gardner , M. Forman , J. Stewart Jr , D. Murray , A. Chancey , Cardiovasc. Res. 2006, 69, 657. 16376324 10.1016/j.cardiores.2005.10.020

[140] N. Pincha , E. Y. Hajam , K. Badarinath , S. P. R. Batta , T. Masudi , R. Dey , P. Andreasen , T. Kawakami , R. Samuel , R. George , D. Danda , P. M. Jacob , C. Jamora , J. Clin. Invest. 2018, 128, 1807. 29584619 10.1172/JCI99088 PMC5919880

[141] S. Moretti , G. Renga , V. Oikonomou , C. Galosi , M. Pariano , R. G. Iannitti , M. Borghi , M. Puccetti , M. De Zuani , C. E. Pucillo , G. Paolicelli , T. Zelante , J.‐C. Renauld , O. Bereshchenko , P. Sportoletti , V. Lucidi , M. C. Russo , C. Colombo , E. Fiscarelli , C. Lass‐Flörl , F. Majo , G. Ricciotti , H. Ellemunter , L. Ratclif , V. N. Talesa , V. Napolioni , L. Romani , Nat. Commun. 2017, 8, 14017. 28090087 10.1038/ncomms14017 PMC5241810

[142] L. Kennedy , V. Meadows , A. Sybenga , J. Demieville , L. Chen , L. Hargrove , B. Ekser , W. Dar , L. Ceci , D. Kundu , K. Kyritsi , L. Pham , T. Zhou , S. Glaser , F. Meng , G. Alpini , H. Francis , Hepatology 2021, 74, 164. 33434322 10.1002/hep.31713 PMC9271361

[143] M. Tsai , P. Valent , S. J. Galli , J. Allergy Clin. Immunol. 2022, 149, 1845. 35469840 10.1016/j.jaci.2022.04.012 PMC9177781

[144] A. Le Moine , V. Flamand , F.‐X. Demoor , J.‐C. Noël , M. Surquin , R. Kiss , M.‐A. Nahori , M. Pretolani , M. Goldman , D. Abramowicz , J. Clin. Invest. 1999, 103, 1659. 10377172 10.1172/JCI5504 PMC408380

[145] A. Solomon , R. Shmilowich , D. Shasha , J. Frucht‐Pery , J. Pe'er , S. Bonini , F. Levi‐Schaffer , Invest. Ophthalmol. Vis. Sci. 2000, 41, 1038. 10752939

[146] I. Gomes , S. K. Mathur , B. M. Espenshade , Y. Mori , J. Varga , S. J. Ackerman , J. Allergy Clin. Immunol. 2005, 116, 796. 16210053 10.1016/j.jaci.2005.06.031

[147] A. Kanda , V. Driss , N. Hornez , M. Abdallah , T. Roumier , G. Abboud , F. Legrand , D. Staumont‐Sallé , S. Quéant , J. Bertout , S. Fleury , P. Rémy , J.‐P. Papin , V. Julia , M. Capron , D. Dombrowicz , J. Allergy Clin. Immunol. 2009, 124, 571. 10.1016/j.jaci.2009.04.031 19539982

[148] Y. Morimoto , K. Hirahara , M. Kiuchi , T. Wada , T. Ichikawa , T. Kanno , M. Okano , K. Kokubo , A. Onodera , D. Sakurai , Y. Okamoto , T. Nakayama , Immunity 2018, 49, 134. 29958800 10.1016/j.immuni.2018.04.023

[149] J. Liu , C. Yang , T. Liu , Z. Deng , W. Fang , X. Zhang , J. Li , Q. Huang , C. Liu , Y. Wang , D. Yang , G. K. Sukhova , J. S. Lindholt , A. Diederichsen , L. M. Rasmussen , D. Li , G. Newton , F. W. Luscinskas , L. Liu , P. Libby , J. Wang , J. Guo , G.‐P. Shi , Nat. Commun. 2020, 11, 6396. 33328477 10.1038/s41467-020-19297-5 PMC7745020

[150] E. Vivier , D. Artis , M. Colonna , A. Diefenbach , J. P. Di Santo , G. Eberl , S. Koyasu , R. M. Locksley , A. N. J. McKenzie , R. E. Mebius , F. Powrie , H. Spits , Cell 2018, 174, 1054. 30142344 10.1016/j.cell.2018.07.017

[151] N. Jacquelot , C. Seillet , E. Vivier , G. T. Belz , Nat. Immunol. 2022, 23, 371. 35228695 10.1038/s41590-022-01127-z

[152] H. Spits , J. H. Bernink , L. Lanier , Nat. Immunol. 2016, 17, 758. 27328005 10.1038/ni.3482

[153] G. M. Jowett , M. D. A. Norman , T. T. L. Yu , P. Rosell Arévalo , D. Hoogland , S. T. Lust , E. Read , E. Hamrud , N. J. Walters , U. Niazi , M. W. H. Chung , D. Marciano , O. S. Omer , T. Zabinski , D. Danovi , G. M. Lord , J. Hilborn , N. D. Evans , C. A. Dreiss , L. Bozec , O. P. Oommen , C. D. Lorenz , R. M. P. da Silva , J. F. Neves , E. Gentleman , Nat. Mater. 2021, 20, 250. 32895507 10.1038/s41563-020-0783-8 PMC7611574

[154] H. Wang , L. Shen , X. Sun , F. Liu , W. Feng , C. Jiang , X. Chu , X. Ye , C. Jiang , Y. Wang , P. Zhang , M. Zang , D. Zhu , Y. Bi , Nat. Commun. 2019, 10, 3254. 31332184 10.1038/s41467-019-11270-1 PMC6646407

[155] A. Melhem , N. Muhanna , A. Bishara , C. E. Alvarez , Y. Ilan , T. Bishara , A. Horani , M. Nassar , S. L. Friedman , R. Safadi , J. Hepatol. 2006, 45, 60. 16515819 10.1016/j.jhep.2005.12.025

[156] C. Gur , S. Doron , S. Kfir‐Erenfeld , E. Horwitz , L. Abu‐tair , R. Safadi , O. Mandelboim , Gut 2012, 61, 885. 22198715 10.1136/gutjnl-2011-301400

[157] S. Varchetta , D. Mele , A. Lombardi , B. Oliviero , S. Mantovani , C. Tinelli , M. Spreafico , D. Prati , S. Ludovisi , G. Ferraioli , C. Filice , A. Aghemo , P. Lampertico , F. Facchetti , F. Bernuzzi , P. Invernizzi , M. U. Mondelli , Gut. 2016, 65, 1998. 26674359 10.1136/gutjnl-2015-310327

[158] X. Tao , R. Zhang , R. Du , T. Yu , H. Yang , J. Li , Y. Wang , Q. Liu , S. Zuo , X. Wang , M. Lazarus , L. Zhou , B. Wang , Y. Yu , Y. Shen , J. Exp. Med. 2022, 219, 20212414. 10.1084/jem.20212414 PMC9014794 35420633

[159] R. S. Wijaya , S. A. Read , S. Schibeci , M. Eslam , M. K. Azardaryany , K. El‐Khobar , D. van der Poorten , R. Lin , L. Yuen , V. Lam , J. George , M. W. Douglas , G. Ahlenstiel , J. Hepatol. 2019, 71, 252. 30905683 10.1016/j.jhep.2019.03.012

[160] W.‐M. Choi , T. Ryu , J.‐H. Lee , Y.‐R. Shim , M.‐H. Kim , H.‐H. Kim , Y. E. Kim , K. Yang , K. Kim , S. E. Choi , W. Kim , S.‐H. Kim , H. S. Eun , W.‐I. Jeong , Hepatology 2021, 74, 2170. 33932306 10.1002/hep.31875

[161] B. Langhans , A. W. Alwan , B. Krämer , A. Glässner , P. Lutz , C. P. Strassburg , J. Nattermann , U. Spengler , J. Hepatol. 2015, 62, 398. 25195554 10.1016/j.jhep.2014.08.038

[162] A. Glässner , M. Eisenhardt , P. Kokordelis , B. Krämer , F. Wolter , H. D. Nischalke , C. Boesecke , T. Sauerbruch , J. K. Rockstroh , U. Spengler , J. Nattermann , J. Hepatol. 2013, 59, 427. 23665286 10.1016/j.jhep.2013.04.029

[163] V. Gonzalez‐Polo , M. Pucci‐Molineris , V. Cervera , S. Gambaro , S. E. Yantorno , V. Descalzi , C. Tiribelli , G. E. Gondolesi , D. Meier , Ann. Hepatol. 2019, 18, 366. 31053540 10.1016/j.aohep.2018.12.001

[164] P. Laurent , B. Allard , P. Manicki , V. Jolivel , E. Levionnois , M. Jeljeli , P. Henrot , J. Izotte , D. Leleu , A. Groppi , J. Seneschal , J. Constans , C. Chizzolini , C. Richez , P. Duffau , E. Lazaro , E. Forcade , T. Schaeverbeke , T. Pradeu , F. Batteux , P. Blanco , C. Contin‐Bordes , M.‐E. Truchetet , Ann. Rheum. Dis. 2021, 80, 1594. 34285051 10.1136/annrheumdis-2020-219748 PMC8600612

[165] J. E. Miller , H. Lingegowda , L. K. Symons , O. Bougie , S. L. Young , B. A. Lessey , M. Koti , C. Tayade , JCI Insight 2021, 6, 149699. 10.1172/jci.insight.149699 PMC8675188 34699382

[166] J. Zhang , J. Qiu , W. Zhou , J. Cao , X. Hu , W. Mi , B. Su , B. He , J. Qiu , L. Shen , Nat. Immunol. 2022, 23, 237. 35075279 10.1038/s41590-021-01097-8

[167] A. Hernandez‐Gutierrez , S. Majid , A. Eberle , A. Choi , P. Sorkhdini , D. Yang , A. X. Yang , C. Norbrun , C. H. He , C.‐M. Lee , C. G. Lee , J. A. Elias , Y. Zhou , J. Clin. Invest. 2023, 133, 169583. 10.1172/JCI169583 PMC10378191 37289545

[168] Y. Nakatsuka , A. Yaku , T. Handa , A. Vandenbon , Y. Hikichi , Y. Motomura , A. Sato , M. Yoshinaga , K. Tanizawa , K. Watanabe , T. Hirai , K. Chin , Y. Suzuki , T. Uehata , T. Mino , T. Tsujimura , K. Moro , O. Takeuchi , Eur. Respir. J. 2021, 57, 2000018. 32978308 10.1183/13993003.00018-2020

[169] A. Altieri , M. B. Buechler , Nat. Rev. Immunol. 2023, 23, 477. 10.1038/s41577-023-00909-2 37369879

[170] Z. Liang , Z. Tang , C. Zhu , F. Li , S. Chen , X. Han , R. Zheng , X. Hu , R. Lin , Q. Pei , C. Yin , J. Wang , C. Tang , N. Cao , J. Zhao , R. Wang , X. Li , N. Luo , Q. Wen , J. Yu , J. Li , X. Xia , X. Zheng , X. Wang , N. Huang , Z. Zhong , C. Mo , P. Chen , Y. Wang , J. Fan , et al., Immunity 2024, 57, 1306. 38815582 10.1016/j.immuni.2024.05.004 PMC11539045

[171] J. Raabe , K. M. Kaiser , M. ToVinh , C. Finnemann , P. Lutz , C. Hoffmeister , J. Bischoff , F. Goeser , D. J. Kaczmarek , T. R. Glowka , S. Manekeller , A. Charpentier , B. Langhans , H. D. Nischalke , M. Toma , C. P. Strassburg , U. Spengler , A. T. Abdallah , B. Krämer , J. Nattermann , Hepatology 2023, 78, 787. 37029085 10.1097/HEP.0000000000000350

[172] B. C. Lo , M. J. Gold , M. R. Hughes , F. Antignano , Y. Valdez , C. Zaph , K. W. Harder , K. M. McNagny , Sci. Immunol. 2016, 1, aaf8864. 10.1126/sciimmunol.aaf8864 PMC5489332 28670633

[173] S. Wang , J. Li , S. Wu , L. Cheng , Y. Shen , W. Ma , W. She , C. Yang , J. Wang , W. Jiang , Clin. Sci. 2018, 132, 2565. 10.1042/CS20180482 30459204

[174] T. Castro‐Dopico , A. Fleming , T. W. Dennison , J. R. Ferdinand , K. Harcourt , B. J. Stewart , Z. Cader , Z. K. Tuong , C. Jing , L. S. C. Lok , R. J. Mathews , A. Portet , A. Kaser , S. Clare , M. R. Clatworthy , Cell Rep. 2020, 32, 107857. 32640223 10.1016/j.celrep.2020.107857 PMC7351110

[175] D. G. Pellicci , H. F. Koay , S. P. Berzins , Nat. Rev. Immunol. 2020, 20, 756. 32581346 10.1038/s41577-020-0345-y

[176] M. G. Constantinides , Y. Belkaid , Science 2021, 374, abf0095. 10.1126/science.abf0095 PMC8697520 34882451

[177] D. I. Godfrey , J. Le Nours , D. M. Andrews , A. P. Uldrich , J. Rossjohn , Immunity 2018, 48, 453. 29562195 10.1016/j.immuni.2018.03.009

[178] C. M. Crosby , M. Kronenberg , Nat. Rev. Immunol. 2018, 18, 559. 29967365 10.1038/s41577-018-0034-2 PMC6343475

[179] M. P. Murray , I. Engel , G. Seumois , S. Herrera‐De la Mata , S. L. Rosales , A. Sethi , A. Logandha Ramamoorthy Premlal , G.‐Y. Seo , J. Greenbaum , P. Vijayanand , J. P. Scott‐Browne , M. Kronenberg , Nat. Commun. 2021, 12, 1446. 33664261 10.1038/s41467-021-21574-w PMC7933435

[180] W. K. Syn , Y. H. Oo , T. A. Pereira , G. F. Karaca , Y. Jung , A. Omenetti , R. P. Witek , S. S. Choi , C. D. Guy , C. M. Fearing , V. Teaberry , F. E. Pereira , D. H. Adams , A. M. Diehl , Hepatology 2010, 51, 1998. 20512988 10.1002/hep.23599 PMC2920131

[181] H. Wang , L. Li , Y. Li , Y. Li , Y. Sha , S. Wen , Q. You , L. Liu , M. Shi , H. Zhou , Theranostics 2021, 11, 2149. 33500717 10.7150/thno.51369 PMC7797696

[182] X. Zhang , P. Sharma , P. Maschmeyer , Y. Hu , M. Lou , J. Kim , H. Fujii , D. Unutmaz , R. F. Schwabe , F. Winau , J. Hepatol. 2023, 79, 1214. 37348791 10.1016/j.jhep.2023.05.043 PMC10592496

[183] H.‐X. Wang , W.‐J. Li , C.‐L. Hou , S. Lai , Y.‐L. Zhang , C. Tian , H. Yang , J. Du , H.‐H. Li , Cardiovasc. Res. 2019, 115, 83. 29939225 10.1093/cvr/cvy164

[184] K. Jandl , L. M. Marsh , A. C. Mutgan , S. Crnkovic , F. Valzano , D. Zabini , J. Hoffmann , V. Foris , E. Gschwandtner , W. Klepetko , H. Prosch , H. Flick , L. Brcic , I. Kern , A. Heinemann , H. Olschewski , G. Kovacs , G. Kwapiszewska , Am J. Respir. Crit. Care Med. 2022, 206, 981. 35763380 10.1164/rccm.202201-0142OC

[185] H. Mehta , M. J. Lett , P. Klenerman , M. Filipowicz Sinnreich , Semin. Immunopathol. 2022, 44, 429. 35641678 10.1007/s00281-022-00949-1 PMC9256577

[186] N. M. Provine , P. Klenerman , Annu. Rev. Immunol. 2020, 38, 203. 31986071 10.1146/annurev-immunol-080719-015428

[187] P. Hegde , E. Weiss , V. Paradis , J. Wan , M. Mabire , S. Sukriti , P.‐E. Rautou , M. Albuquerque , O. Picq , A. C. Gupta , G. Ferrere , H. Gilgenkrantz , B. Kiaf , A. Toubal , L. Beaudoin , P. Lettéron , R. Moreau , A. Lehuen , S. Lotersztajn , Nat. Commun. 2018, 9, 2146. 29858567 10.1038/s41467-018-04450-y PMC5984626

[188] M. Mabire , P. Hegde , A. Hammoutene , J. Wan , C. Caër , R. A. Sayegh , M. Cadoux , M. Allaire , E. Weiss , T. Thibault‐Sogorb , O. Lantz , M. Goodhardt , V. Paradis , P. de la Grange , H. Gilgenkrantz , S. Lotersztajn , Nat. Commun. 2023, 14, 1830. 37005415 10.1038/s41467-023-37453-5 PMC10067815

[189] B. M. P. Law , R. Wilkinson , X. Wang , K. Kildey , K. Giuliani , K. W. Beagley , J. Ungerer , H. Healy , A. J. Kassianos , J. Am. Soc. Nephrol. 2019, 30, 1322. 31186283 10.1681/ASN.2018101064 PMC6622420

[190] J. C. Ribot , N. Lopes , B. Silva‐Santos , Nat. Rev. Immunol. 2021, 21, 221. 33057185 10.1038/s41577-020-00452-4

[191] S. Marinovic , M. Lenartic , K. Mladenic , M. Sestan , I. Kavazovic , A. Benic , M. Krapic , L. Rindlisbacher , M. Cokaric Brdovcak , C. Sparano , G. Litscher , T. Turk Wensveen , I. Mikolasevic , D. Fuckar Cupic , L. Bilic‐Zulle , A. Steinle , A. Waisman , A. Hayday , S. Tugues , B. Becher , B. Polic , F. M. Wensveen , Sci. Immunol. 2023, 8, add1599. 10.1126/sciimmunol.add1599 PMC7615627 37774007

[192] X. Peng , Z. Xiao , J. Zhang , Y. Li , Y. Dong , J. Du , J. Pathol. 2015, 235, 79. 25158055 10.1002/path.4430

[193] B. M.‐P. Law , R. Wilkinson , X. Wang , K. Kildey , M. Lindner , K. Beagley , H. Healy , A. J. Kassianos , Nephrol., Dial., Transplant. 2019, 34, 40. 29897565 10.1093/ndt/gfy098

[194] Q. Liu , Q. Yang , Z. Wu , Y. Chen , M. Xu , H. Zhang , J. Zhao , Z. Liu , Z. Guan , J. Luo , Z. Y. Li , G. Sun , Q. Wen , Y. Xu , Z. Li , K. Chen , X. Ben , W. He , X. Li , Z. Yin , J. Hao , L. Lu , Cell Death Dis. 2022, 13, 289. 35361750 10.1038/s41419-022-04739-3 PMC8971410

[195] P. L. Simonian , F. Wehrmann , C. L. Roark , W. K. Born , R. L. O'Brien , J. Exp. Med. 2010, 207, 2239. 20855496 10.1084/jem.20100061 PMC2947077

[196] H. Shen , L. Sheng , Y. Xiong , Y.‐H. Kim , L. Jiang , Z. Chen , Y. Liu , K. Pyaram , C.‐H. Chang , L. Rui , J. Hepatol. 2017, 67, 100. 28267623 10.1016/j.jhep.2017.02.025 PMC5476485

[197] F. do Valle Duraes , A. Lafont , M. Beibel , K. Martin , K. Darribat , R. Cuttat , A. Waldt , U. Naumann , G. Wieczorek , S. Gaulis , S. Pfister , K. D. Mertz , J. Li , G. Roma , M. Warncke , JCI Insight 2020, 5, 130651. 32051345 10.1172/jci.insight.130651 PMC7098794

[198] J. Glaubitz , A. Wilden , J. Golchert , G. Homuth , U. Völker , B. M. Bröker , T. Thiele , M. M. Lerch , J. Mayerle , A. A. Aghdassi , F. U. Weiss , M. Sendler , Nat. Commun. 2022, 13, 4502. 35922425 10.1038/s41467-022-32195-2 PMC9349313

[199] J. Wen , Y. Zhou , J. Wang , J. Chen , W. Yan , J. Wu , J. Yan , K. Zhou , Y. Xiao , Y. Wang , Q. Xia , W. Cai , Cell Death Differ. 2017, 24, 997. 28304404 10.1038/cdd.2017.31 PMC5442468

[200] T. Nevers , A. M. Salvador , F. Velazquez , N. Ngwenyama , F. J. Carrillo‐Salinas , M. Aronovitz , R. M. Blanton , P. Alcaide , J. Exp. Med. 2017, 214, 3311. 28970239 10.1084/jem.20161791 PMC5679176

[201] J. Jimenez , J. Amrute , P. Ma , X. Wang , S. Das , R. Dai , Y. Komaru , A. Herrlich , M. Mack , K. J. Lavine , Nat. Cardiovasc. Res. 2025, 4, 458. 40217124 10.1038/s44161-025-00633-1 PMC12641230

[202] P. Ma , J. Liu , J. Qin , L. Lai , G. S. Heo , H. Luehmann , D. Sultan , A. Bredemeyer , G. Bajapa , G. Feng , J. Jimenez , R. He , A. Parks , J. Amrute , A. Villanueva , Y. Liu , C.‐Y. Lin , M. Mack , K. Amancherla , J. Moslehi , K. J. Lavine , Circulation 2024, 149, 48. 37746718 10.1161/CIRCULATIONAHA.122.062551 PMC11323830

[203] G. C. Baldeviano , J. G. Barin , M. V. Talor , S. Srinivasan , D. Bedja , D. Zheng , K. Gabrielson , Y. Iwakura , N. R. Rose , D. Cihakova , Circ. Res. 2010, 106, 1646. 20378858 10.1161/CIRCRESAHA.109.213157

[204] Y. Koda , T. Teratani , P. S. Chu , Y. Hagihara , Y. Mikami , Y. Harada , H. Tsujikawa , K. Miyamoto , T. Suzuki , N. Taniki , T. Sujino , M. Sakamoto , T. Kanai , N. Nakamoto , Nat. Commun. 2021, 12, 4474. 34294714 10.1038/s41467-021-24734-0 PMC8298513

[205] L. Zhang , C. Zhao , W. Dai , H. Tong , W. Yang , Z. Huang , C. Tang , J. Gao , Cell. Mol. Life Sci. 2023, 80, 379. 38010435 10.1007/s00018-023-05032-y PMC11072584

[206] C. M. Prêle , T. Miles , D. R. Pearce , R. J. O'Donoghue , C. Grainge , L. Barrett , K. Birnie , A. D. Lucas , S. Baltic , M. Ernst , C. Rinaldi , G. J. Laurent , D. A. Knight , M. Fear , G. Hoyne , R. J. McAnulty , S. E. Mutsaers , Eur. Respir. J. 2022, 60, 2101469. 35798357 10.1183/13993003.01469-2021 PMC9684624

[207] F. Barrow , S. Khan , G. Fredrickson , H. Wang , K. Dietsche , P. Parthiban , S. Robert , T. Kaiser , S. Winer , A. Herman , O. Adeyi , M. Mouzaki , A. Khoruts , K. A. Hogquist , C. Staley , D. A. Winer , X. S. Revelo , Hepatology 2021, 74, 704. 33609303 10.1002/hep.31755 PMC8377092

[208] T. Matsushita , T. Kobayashi , K. Mizumaki , M. Kano , T. Sawada , M. Tennichi , A. Okamura , Y. Hamaguchi , Y. Iwakura , M. Hasegawa , M. Fujimoto , K. Takehara , Sci. Adv. 2018, 4, aas9944. 10.1126/sciadv.aas9944 PMC6040844 30009261

[209] H. Numajiri , A. Kuzumi , T. Fukasawa , S. Ebata , A. Yoshizaki‐Ogawa , Y. Asano , Y. Kazoe , K. Mawatari , T. Kitamor , Arthritis Rheumatol. 2021, 73, 2086. 33955200 10.1002/art.41798

[210] F. Faggioli , E. Palagano , L. Di Tommaso , M. Donadon , V. Marrella , C. Recordati , S. Mantero , A. Villa , P. Vezzoni , B. Cassani , Hepatology 2018, 67, 1970. 29105104 10.1002/hep.29636

[211] J. Jiao , S. He , Y. Wang , Y. Lu , M. Gu , D. Li , T. Tang , S. Nie , M. Zhang , B. Lv , J. Li , N. Xia , X. Cheng , Basic Res. Cardiol. 2021, 116, 46. 34302556 10.1007/s00395-021-00886-4 PMC8310480

[212] K. Oleinika , E. C. Rosser , D. E. Matei , K. Nistala , A. Bosma , I. Drozdov , C. Mauri , Nat. Commun. 2018, 9, 684. 29449556 10.1038/s41467-018-02911-y PMC5814456

[213] L. Zhang , J. Tian , N. Li , Y. Wang , Y. Jin , H. Bian , M. Xiong , Z. Zhang , J. Meng , Z. Han , S. Duan , J. Hazard. Mater. 2025, 483, 136629. 39603130 10.1016/j.jhazmat.2024.136629

[214] F. S. Younesi , A. E. Miller , T. H. Barker , F. M. V. Rossi , B. Hinz , Nat. Rev. Mol. Cell Biol. 2024, 25, 617. 38589640 10.1038/s41580-024-00716-0

[215] F. Rieder , S. P. Kessler , G. A. West , S. Bhilocha , C. de la Motte , T. M. Sadler , B. Gopalan , E. Stylianou , C. Fiocchi , Am. J. Pathol. 2011, 179, 2660. 21945322 10.1016/j.ajpath.2011.07.042 PMC3204019

[216] R. Mothes , A. Pascual‐Reguant , R. Koehler , J. Liebeskind , A. Liebheit , S. Bauherr , L. Philipsen , C. Dittmayer , M. Laue , R. von Manitius , S. Elezkurtaj , P. Durek , F. Heinrich , G. A. Heinz , G. M. Guerra , B. Obermayer , J. Meinhardt , J. Ihlow , J. Radke , F. L. Heppner , P. Enghard , H. Stockmann , T. Aschman , J. Schneider , V. M. Corman , L. E. Sander , M.‐F. Mashreghi , T. Conrad , A. C. Hocke , R. A. Niesner , et al., Nat. Commun. 2023, 14, 791. 36774347 10.1038/s41467-023-36333-2 PMC9922044

[217] A. Niculae , M. E. Gherghina , I. Peride , M. Tiglis , A. M. Nechita , I. A. Checherita , Int. J. Mol. Sci. 2023, 24, 14019. 37762322 10.3390/ijms241814019 PMC10531003

[218] H. Zhao , World J. Nephrol. 2013, 2, 84.24255890

[219] J. U. Igietseme , Y. Omosun , O. Stuchlik , M. S. Reed , J. Partin , Q. He , K. Joseph , D. Ellerson , B. Bollweg , Z. George , F. O. Eko , C. Bandea , H. Liu , G. Yang , W.‐J. Shieh , J. Pohl , K. Karem , C. M. Black , PLoS One 2015, 10, 0145198. 10.1371/journal.pone.0145198 PMC4683008 26681200

[220] D. Ma , Y. Feng , X. Lin , Front. Immunol. 2024, 15, 1421436. 39469708 10.3389/fimmu.2024.1421436 PMC11513355

[221] Y. Dong , M. Yang , J. Zhang , X. Peng , J. Cheng , T. Cui , J. Du , J. Immunol. 2016, 196, 1874. 26773152 10.4049/jimmunol.1501232

[222] B. Liu , J. Jiang , H. Liang , P. Xiao , X. Lai , J. Nie , W. Yu , Y. Gao , S. Wen , Int. Immunopharmacol. 2021, 98, 107907. 34243040 10.1016/j.intimp.2021.107907

[223] X. Di , Y. Li , J. Wei , T. Li , B. Liao , Adv. Sci. 2025, 12, 2410416. 10.1002/advs.202410416 PMC11744640 39665319

[224] T. E. King , W. Z. Bradford , S. Castro‐Bernardini , E. A. Fagan , I. Glaspole , M. K. Glassberg , E. Gorina , P. M. Hopkins , D. Kardatzke , L. Lancaster , D. J. Lederer , S. D. Nathan , C. A. Pereira , S. A. Sahn , R. Sussman , J. J. Swigris , P. W. Noble , N. Engl. J. Med. 2014, 370, 2083. 24836312 10.1056/NEJMoa1402582

[225] L. Richeldi , R. M. du Bois , G. Raghu , A. Azuma , K. K. Brown , U. Costabel , V. Cottin , K. R. Flaherty , D. M. Hansell , Y. Inoue , D. S. Kim , M. Kolb , A. G. Nicholson , P. W. Noble , M. Selman , H. Taniguchi , M. Brun , F. Le Maulf , M. Girard , S. Stowasser , R. Schlenker‐Herceg , B. Disse , H. R. Collard , N. Engl. J. Med. 2014, 370, 2071.24836310

[226] S. A. Harrison , P. Bedossa , C. D. Guy , J. M. Schattenberg , R. Loomba , R. Taub , D. Labriola , S. E. Moussa , G. W. Neff , M. E. Rinella , Q. M. Anstee , M. F. Abdelmalek , Z. Younossi , S. J. Baum , S. Francque , M. R. Charlton , P. N. Newsome , N. Lanthier , I. Schiefke , A. Mangia , J. M. Pericàs , R. Patil , A. J. Sanyal , M. Noureddin , M. B. Bansal , N. Alkhouri , L. Castera , M. Rudraraju , V. Ratziu , N. Engl. J. Med. 2024, 390, 497. 38324483 10.1056/NEJMoa2309000

[227] A. Tefferi , D. Barraco , T. L. Lasho , S. Shah , K. H. Begna , A. Al‐Kali , W. J. Hogan , M. R. Litzow , C. A. Hanson , R. P. Ketterling , N. Gangat , A. Pardanani , Blood Cancer J. 2018, 8, 29. 29515114 10.1038/s41408-018-0067-6 PMC5841331

[228] A. Pardanani , J. Gotlib , A. W. Roberts , M. Wadleigh , S. Sirhan , J. Kawashima , J. A. Maltzman , L. Shao , V. Gupta , A. Tefferi , Leukemia 2018, 32, 1034. 10.1038/leu.2017.330 29263442

[229] J. H. W. Distler , A. H. Gyorfi , M. Ramanujam , M. L. Whitfield , M. Konigshoff , R. Lafyatis , Nat. Rev. Rheumatol. 2019, 15, 705. 31712723 10.1038/s41584-019-0322-7

[230] D. N. O'Dwyer , S. L. Ashley , B. B. Moore , Am. J. Physiol. Lung Cell. Mol. Physiol. 2016, 311, L590. 27474089 10.1152/ajplung.00221.2016 PMC5142210

[231] J. D. Chalmers , C. S. Haworth , M. L. Metersky , M. R. Loebinger , F. Blasi , O. Sibila , A. E. O'Donnell , E. J. Sullivan , K. C. Mange , C. Fernandez , J. Zou , C. L. Daley , N. Engl. J. Med. 2020, 383, 2127. 32897034 10.1056/NEJMoa2021713

[232] N. Mahtal , O. Lenoir , C. Tinel , D. Anglicheau , P. L. Tharaux , Nat. Rev. Nephrol. 2022, 18, 643. 35974169 10.1038/s41581-022-00608-6

[233] D. Tampe , M. Zeisberg , Nat. Rev. Nephrol. 2014, 10, 226. 24514753 10.1038/nrneph.2014.14

[234] J. G. Rurik , I. Tombácz , A. Yadegari , P. O. Méndez Fernández , S. V. Shewale , L. Li , T. Kimura , O. Y. Soliman , T. E. Papp , Y. K. Tam , B. L. Mui , S. M. Albelda , E. Puré , C. H. June , H. Aghajanian , D. Weissman , H. Parhiz , J. A. Epstein , Science 2022, 375, 91. 34990237 10.1126/science.abm0594 PMC9983611

[235] C. Amor , J. Feucht , J. Leibold , Y.‐J. Ho , C. Zhu , D. Alonso‐Curbelo , J. Mansilla‐Soto , J. A. Boyer , X. Li , T. Giavridis , A. Kulick , S. Houlihan , E. Peerschke , S. L. Friedman , V. Ponomarev , A. Piersigilli , M. Sadelain , S. W. Lowe , Nature 2020, 583, 127. 32555459 10.1038/s41586-020-2403-9 PMC7583560

[236] H. Dai , C. Zhu , Q. Huai , W. Xu , J. Zhu , X. Zhang , X. Zhang , B. Sun , H. Xu , M. Zheng , X. Li , H. Wang , J. Hepatol. 2024, 80, 913. 38340812 10.1016/j.jhep.2024.01.034

[237] J. Wang , H. Du , W. Xie , J. Bi , H. Zhang , X. Liu , Y. Wang , S. Zhang , A. Lei , C. He , H. Yuan , J. Zhang , Y. Li , P. Xu , S. Liu , Y. Zhou , J. Shen , J. Wu , Y. Cai , C. Yang , Z. Li , Y. Liang , Y. Zhao , J. Zhang , M. Song , Circ. Res. 2024, 135, 1161. 39465245 10.1161/CIRCRESAHA.124.325212

[238] T. Hu , A. R. Kumar , Y. Luo , A. Tay , Small Methods 2024, 8, 2301300. 10.1002/smtd.202301300 38054597

[239] T. Chen , J. Deng , Y. Zhang , B. Liu , R. Liu , Y. Zhu , M. Zhou , Y. Lin , B. Xia , K. Lin , X. Ma , H. Zhang , Mol. Cancer 2024, 23, 53. 38468291 10.1186/s12943-024-01938-8 PMC10926606

[240] Y. Zhang , F. Zhou , Z. Wu , Y. Li , C. Li , M. Du , W. Luo , H. Kou , C. Lu , H. Mei , Front. Immunol. 2022, 13, 914959. 35799791 10.3389/fimmu.2022.914959 PMC9253384

[241] C. W. Freyer , D. L. Porter , J. Allergy Clin. Immunol. 2020, 146, 940. 32771558 10.1016/j.jaci.2020.07.025

[242] Y. Wang , A. Buck , B. Piel , L. Zerefa , N. Murugan , C. D. Coherd , A. G. Miklosi , H. Johal , R. N. Bastos , K. Huang , M. Ficial , Y. N. Laimon , S. Signoretti , Z. Zhong , S.‐M. Hoang , G. M. Kastrunes , M. Grimaud , A. Fayed , H.‐C. Yuan , Q.‐D. Nguyen , T. Thai , E. V. Ivanova , C. P. Paweletz , M.‐R. Wu , T. K. Choueiri , J. O. Wee , G. J. Freeman , D. A. Barbie , W. A. Marasco , Mol. Cancer 2024, 23, 56. 38491381 10.1186/s12943-024-01952-w PMC10943873

[243] S. Fanucchi , J. Dominguez‐Andres , L. A. B. Joosten , M. G. Netea , M. M. Mhlanga , Immunity 2021, 54, 32. 33220235 10.1016/j.immuni.2020.10.011

[244] M. G. Netea , J. Domínguez‐Andrés , L. B. Barreiro , T. Chavakis , M. Divangahi , E. Fuchs , L. A. B. Joosten , J. W. M. van der Meer , M. M. Mhlanga , W. J. M. Mulder , N. P. Riksen , A. Schlitzer , J. L. Schultze , C. Stabell Benn , J. C. Sun , R. J. Xavier , E. Latz , Nat. Rev. Immunol. 2020, 20, 375. 32132681 10.1038/s41577-020-0285-6 PMC7186935

[245] R. Kamada , W. Yang , Y. Zhang , M. C. Patel , Y. Yang , R. Ouda , A. Dey , Y. Wakabayashi , K. Sakaguchi , T. Fujita , T. Tamura , J. Zhu , K. Ozato , Proc. Natl. Acad. Sci. U. S. A. 2018, 115, E9162. 30201712 10.1073/pnas.1720930115 PMC6166839

[246] F. Saaoud , L. Liu , K. Xu , R. Cueto , Y. Shao , Y. Lu , Y. Sun , N. W. Snyder , S. Wu , L. Yang , Y. Zhou , D. L. Williams , C. Li , L. Martinez , R. I. Vazquez‐Padron , H. Zhao , X. Jiang , H. Wang , X. Yang , JCI Insight 2023, 8, 158183. 10.1172/jci.insight.158183 PMC9870092 36394956

[247] H. Chen , J. Song , L. Zeng , J. Zha , J. Zhu , A. Chen , Y. Liu , Z. Dong , G. Chen , Biochem. Pharmacol. 2024, 229, 116505. 39181336 10.1016/j.bcp.2024.116505

[248] A. Simats , S. Zhang , D. Messerer , F. Chong , S. Beskardes , A. S. Chivukula , J. Cao , S. Besson‐Girard , F. A. Montellano , C. Morbach , O. Carofiglio , A. Ricci , S. Roth , G. Llovera , R. Singh , Y. Chen , S. Filser , N. Plesnila , C. Braun , H. Spitzer , O. Gokce , M. Dichgans , P. U. Heuschmann , K. Hatakeyama , E. Beltrán , S. Clauss , B. Bonev , C. Schulz , A. Liesz , Cell 2024, 187, 4637. 39043180 10.1016/j.cell.2024.06.028

[249] Y. Nakayama , K. Fujiu , T. Oshima , J. Matsuda , J. Sugita , T. J. Matsubara , Y. Liu , K. Goto , K. Kani , R. Uchida , N. Takeda , H. Morita , Y. Xiao , M. Hayashi , Y. Maru , E. Hasumi , T. Kojima , S. Ishiguro , Y. Kijima , N. Yachie , S. Yamazaki , R. Yamamoto , F. Kudo , M. Nakanishi , A. Iwama , R. Fujiki , A. Kaneda , O. Ohara , R. Nagai , I. Manabe , et al., Sci. Immunol. 2024, 9, ade3814. 10.1126/sciimmunol.ade3814 38787963

[250] Y.‐Y. Kang , D.‐Y. Kim , S.‐Y. Lee , H.‐J. Kim , T. Kim , J. A. Cho , T. Lee , E. Y. Choi , Adv. Sci. 2024, 11, 2308978. 10.1002/advs.202308978 PMC11005705 38279580

[251] X. Li , Q. Huai , C. Zhu , X. Zhang , W. Xu , H. Dai , H. Wang , Cell Mol. Gastroenterol. Hepatol. 2023, 16, 711. 37499753 10.1016/j.jcmgh.2023.07.009 PMC10520366

[252] P. Horn , F. Tacke , Cell Metab. 2024, 36, 1439. 38823393 10.1016/j.cmet.2024.05.003

[253] F. Xu , M. Guo , W. Huang , L. Feng , J. Zhu , K. Luo , J. Gao , B. Zheng , L.‐D. Kong , T. Pang , X. Wu , Q. Xu , Redox Biol. 2020, 36, 101634. 32863213 10.1016/j.redox.2020.101634 PMC7369618

[254] Q. Kong , N. Li , H. Cheng , X. Zhang , X. Cao , T. Qi , L. Dai , Z. Zhang , X. Chen , C. Li , Y. Li , B. Xue , L. Fang , L. Liu , Z. Ding , Diabetes 2019, 68, 361. 30455376 10.2337/db18-0035

[255] J. Rao , H. Wang , M. Ni , Z. Wang , Z. Wang , S. Wei , M. Liu , P. Wang , J. Qiu , L. Zhang , C. Wu , H. Shen , X. Wang , F. Cheng , L. Lu , Gut 2022, 71, 2539. 35140065 10.1136/gutjnl-2021-325150 PMC9664121

[256] X.‐F. Lin , X.‐N. Cui , J. Yang , Y.‐F. Jiang , T.‐J. Wei , L. Xia , X.‐Y. Liao , F. Li , D.‐D. Wang , J. Li , Q. Wu , D.‐S. Yin , Y.‐Y. Le , K. Yang , R. Wei , T.‐P. Hong , Acta Pharmacol. Sin. 2024, 45, 2579. 39294445 10.1038/s41401-024-01389-3 PMC11579449

[257] S. Zhang , Y. Zhang , X. Duan , B. Wang , Z. Zhan , Circulation 2024, 149, 1982. 38390737 10.1161/CIRCULATIONAHA.123.065506 PMC11175795

[258] Y. Jia , J. Chen , Z. Zheng , Y. Tao , S. Zhang , M. Zou , Y. Yang , M. Xue , F. Hu , Y. Li , Q. Zhang , Y. Xue , Z. Zheng , Mol. Med. 2022, 28, 95. 35962319 10.1186/s10020-022-00525-1 PMC9373297

[259] J. Sun , M. Wu , L. Wang , P. Wang , T. Xiao , S. Wang , Q. Liu , Ecotoxicol. Environ. Saf. 2022, 248, 114321. 36427370 10.1016/j.ecoenv.2022.114321

[260] M. Schilperoort , D. Ngai , M. Katerelos , D. A. Power , I. Tabas , Nat. Metab. 2023, 5, 431. 36797420 10.1038/s42255-023-00736-8 PMC10050103

[261] C. Ye , J. Zhu , J. Wang , D. Chen , L. Meng , Y. Zhan , R. Yang , S. He , Z. Li , S. Dai , Y. Li , S. Sun , Z. Shen , Y. Huang , R. Dong , G. Chen , S. Zheng , Clin. Transl. Med. 2022, 12, 1070. 10.1002/ctm2.1070 PMC9636046 36333281

[262] T. Doke , A. Abedini , D. L. Aldridge , Y.‐W. Yang , J. Park , C. M. Hernandez , M. S. Balzer , R. Shrestra , G. Coppock , J. M. I. Rico , S. Y. Han , J. Kim , S. Xin , A. M. Piliponsky , M. Angelozzi , V. Lefebvre , M. C. Siracusa , C. A. Hunter , K. Susztak , Nat. Immunol. 2022, 23, 947. 35552540 10.1038/s41590-022-01200-7 PMC11783796

[263] L. Du , X. Sun , H. Gong , T. Wang , L. Jiang , C. Huang , X. Xu , Z. Li , H. Xu , L. Ma , W. Li , T. Chen , Q. Xu , Stem Cell Res. Ther. 2023, 14, 33. 36805782 10.1186/s13287-023-03256-0 PMC9942332

